# Pharmacokinetics, Tissue Distribution, and Excretion Characteristics of a Radix Polygoni Multiflori Extract in Rats

**DOI:** 10.3389/fphar.2022.827668

**Published:** 2022-02-21

**Authors:** Wenhao Cheng, Siyang Wu, Zheng Yuan, Weiyu Hu, Xin Yu, Nianxin Kang, Qiutao Wang, Mingying Zhu, Kexin Xia, Wei Yang, Chen Kang, Shuofeng Zhang, Yingfei Li

**Affiliations:** ^1^ School of Chinese Pharmacy, Beijing University of Chinese Medicine, Beijing, China; ^2^ Center for DMPK Research of Herbal Medicines, Institute of Chinese Materia Medica, China Academy of Chinese Medical Sciences, Beijing, China; ^3^ Department of Hepatobiliary Pancreatic Surgery, The Affiliated Hospital of Qingdao University, Qingdao, China; ^4^ School of Life Sciences, Beijing University of Chinese Medicine, Beijing, China; ^5^ School of Traditional Chinese Medicine, Shandong University of Traditional Chinese Medicine, Jinan, China

**Keywords:** Radix Polygoni Multiflori, pharmacokinetics, tissue distribution, excretion, UPLC-MS/MS

## Abstract

Although progress has been achieved in the pharmacological activity and toxicity of Radix Polygoni Multiflori (RPM), the chemical basis of its toxicity is still unclear. Here, we performed a multicompound pharmacokinetic analysis and investigated the tissue distribution and excretion characteristics of RPM components after oral administration in rats. The findings demonstrated that the active ingredients of the RPM extract were quickly absorbed after oral administration, with high exposure levels of emodin, 2,3,5,4′-teterahydroxystilbene-2-O-β-D-glucoside (TSG), citreorosein, torachrysone-8-O-glucoside (TG), emodin-8-O-β-D-glucoside (EG), and physcion-8-O-β-D-glucoside (PG). The tissue distributions of emodin, TSG, TG, EG, and PG were high in the liver and kidney. These components were the key contributors to the effectiveness and toxicity of RPM on the liver and kidney. Most of the active ingredients were mainly excreted through feces and bile, while a few were converted into other products in the body and excreted through urine and feces.

## 1 Introduction

Radix Polygoni Multiflori (RPM), the dried tuberous roots of *Reynoutria multiflora* (Thunb.) Moldenke (Polygonaceae), is a popular traditional Chinese medicine that has been used for hair darkening, prolonging life, and mitigating several dysfunctions (Tang et al., 2017). Modern pharmacological research and clinical practices have shown that RPM has several bioactive properties, including antioxidant (Liu X. et al., 2021; Wang et al., 2008), antiaging (Cheung et al., 2014; Fan et al., 2021), and antitumor activities (Ma et al., 2012; Hou et al., 2013; Way et al., 2014; Lin et al., 2017; Xing et al., 2019), and it helps in regulating immunity (Chen et al., 2012), lowering blood lipids (Yao et al., 2013; Chang et al., 2016; Xie et al., 2012), and promoting neuroprotection (Sun et al., 2011; Yang X.-P. et al., 2014; Ruan et al., 2021). However, an increasing number of recently published studies have demonstrated the adverse effects of RPM. For instance, some studies have demonstrated that RPM exerts hepatotoxicity and shows possible drug interactions with warfarin, thus leading to bone marrow suppression (Lin et al., 2015; Liu et al., 2019).

To date, over 100 bioactive constituents of RPM have been isolated and identified, including stilbenes, anthraquinones, flavonoids, and phenolic acids. Of these, stilbene glycosides and anthraquinones are the main active or toxic components of RPM. Despite several reports on the toxicology of RPM, comprehensive information is not available on the chemical basis and mechanism of its toxicity. An herbal constituent can be defined as drug-like if it possesses the desired pharmacologic potency, a wide safety margin, appropriate pharmacokinetic (PK) properties, and adequate content in the prescribed dosage (Jia et al., 2015). TSG is one of the main active components in RPM, and it has strong antioxidant (Büchter et al., 2015) and antiatherosclerotic abilities (Yao et al., 2015; Qian et al., 2020), and can prevent thromboembolic diseases by inhibiting the proliferation of vascular smooth muscle (Xu et al., 2012; Xiang et al., 2014). Emodin can inhibit glutamate-induced apoptosis, and it has a significant neuroprotective effect (Ahn et al., 2016) and can also delay atherosclerotic plaque formation in gene knockout mice through the JAK2-STST3 pathway (Mei-Juan et al., 2018). Both physcion and questin can inhibit the growth of human colon cancer cells by inhibiting Cdc25B phosphatase (Choi et al., 2007). EG and PG can inhibit the activity of soluble epoxide hydrolase and thus have potential antitumor activity (Kwon et al., 2009; Sun et al., 2015), while TG can promote the proliferation of dermal papilla cells and significantly increase hair length (Wu et al., 2018). Rhein protects the liver and exerts antifibrotic effects, and its mechanism may be related to its anti-inflammatory and antioxidant effects and ability to inhibit transforming growth factor β1 and hepatic stellate cell activation (En-Ze et al., 2016). Chrysophanol can inhibit the growth of HeLa cervical cancer cells (Trybus et al., 2021). Citreorosein suppresses the gene expression of proinflammatory cytokines, including tumor necrosis factor (TNF)-α, interleukin (IL)-6 and IL-1β, in mouse bone marrow-derived mast cells (BMMCs) stimulated with phorbol 12-myristate 13-acetate (PMA) plus the calcium ionophore A23187 (Lu et al., 2012). Questinol can significantly inhibit NO production at the indicated concentrations and has also been found to inhibit the production of proinflammatory cytokines, including TNF-α and IL-1β (Yang X. et al., 2014), while CG at low concentrations (12 and 24 μM) significantly increase L-02 cell viability (Liu et al., 2018). These pharmacological effects are consistent with the pharmacological antioxidative, antiaging, neuroprotective, antitumor and anti-inflammatory effects of RPM and may all be effective components in RPM (Lin et al., 2015). RPM can cause liver injury and has certain hepatotoxicity, which is mostly believed to be caused by anthraquinone compounds. Studies have pointed out that rhein, emodin and PG in Polygonum multiflorum have certain cytotoxic effects on HepG2 cells, while rhein, emodin, chrysophanol, aloe emodin, EG and CG have inhibitory effects on HepG2 cells at high concentrations (Yang et al., 2016). TSG compounds may also be related to the hepatotoxicity of Polygonum multiflorum (Lin et al., 2015; Lv et al., 2015).

The pharmacokinetic characteristics of the active ingredients in traditional Chinese medicine have been used to predict the efficacy and potential toxicity of medicines and guide the rational clinical use of drugs (Wang et al., 2013; Zhang et al., 2018). Therefore, pharmacokinetic studies of the main bioactive components in RPM are essential for promoting their clinical application and may help clarify their pharmacological or toxicological action. However, pharmacokinetic studies of RPM mainly focus on its single component and single-dose components, and only a single study has analyzed the pharmacokinetics of seven components in RPM. In addition, although tissue distribution and excretion studies are important in understanding the mechanisms of action of a bioactive compound to the best of our knowledge, RPM has not been fully explored. Therefore, we hypothesized that a comprehensive knowledge of the absorption, distribution and excretion processes of RPM could provide insights on the pharmacodynamic and toxicological chemical basis of its toxicity.

This study aimed to investigate the pharmacokinetics, tissue distribution, and excretion of RPM in rats after oral administration. Using an established ultra-performance liquid chromatography-tandem mass spectrometry (UPLC-MS/MS) procedure (Cheng et al., 2020), several biological components in rat plasma were simultaneously measured, and the bioactive constituents of’ RPM in rat plasma were obtained. In addition, the tissue distribution and excretion routes of RPM ingredients in rats following an oral dose of RPM extract were also elucidated. This study provides helpful references for developing and utilizing the effective ingredients in RPM.

## 2 Materials and Methods

### 2.1 Chemicals and Reagents

**FIGURE 1:**
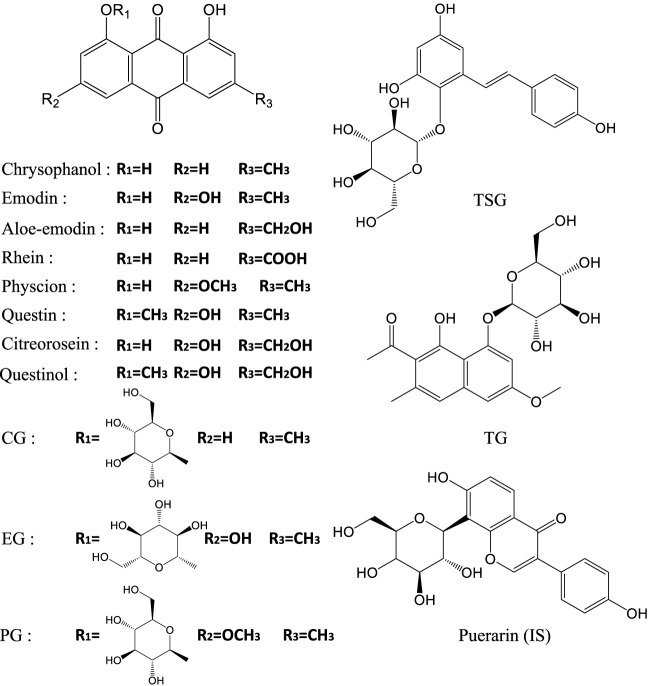
The structures of the 13 constituents of RPM and intenal standard.

The dried herbal roots of *Reynoutria multiflora* (Thunb.) Moldenke (Polygonaceae) (RPM) were purchased from Beijing Tongrentang Pharmaceutical Co., Ltd. The raw materials were carefully authenticated by one of the authors, Prof. Shuofeng Zhang. The voucher specimen (lot no: YDZY-HSW-20180521) was deposited in a refrigerator at 4°C. Chrysophanol, emodin, aloe-emodin, rhein, physcion, questin, emodin-8-O-β-D-glucoside (EG), physcion-8-O-β-D-glucoside (PG), 2,3,5,4′-teterahydroxystilbene-2-O-β-D-glucoside (TSG), and puerarin (IS) were obtained from Chengdu Chroma-Biotechnology Co., Ltd. (Chengdu, China). Citreorosein, questinol, torachrysone-8-O-glucoside (TG), and chrysophanol-8-O-β-D-glucoside (CG) were purchased from Qingdao Advance Chem Technology Co., Ltd. (Qingdao, China). The purity of the various compounds was 98% (Figure 1). Sodium carboxymethyl cellulose (CMC-Na) was purchased from Sinopharm Chemical Reagent Co., Ltd (Shanghai, China). LC-grade acetonitrile and formic acid were purchased from Honeywell (Morristown, United States) and Roe Scientific Inc. (Newark, United States), respectively. Ultra-pure water was prepared using a Millipore Milli-Q purification system (Bedford, United States).

### 2.2 Animals

Sprague–Dawley rats (200–240 g) were obtained from Beijing Vital River Laboratory Animal Technology Co., Ltd. (Beijing, China) [SCXK 2016 (jing)-0006]. The animals were maintained in a specific pathogen-free animal room maintained at 22 ± 2°C, 60 ± 5% humidity and a 12/12 h day/night cycle for at least 7 days prior to the study. The animals had free access to demineralized water and diets, with all nutrients at the standard levels. Before the experiment, the rats were fasted overnight for 12 h and were allowed free access to food 4 h after administration. Both before and during the experiment, water was provided *ad libitum*. The surgical rats were allowed to regain their preoperative body weight prior to the study, and they were euthanized with CO_2_ gas after the study. The experimental protocol (2020B110) was approved by the experimental animal welfare ethics committee at the Institute of Chinese Materia Medica, China Academy of Chinese Medical Sciences (Beijing, China) and performed in accordance with the “Laboratory animal—Guideline for ethical review of animal welfare” (GB/T 35, 892–2018) issued by the General Administration of Quality Supervision, Inspection and Quarantine of the People’s Republic of China (2018).

### 2.3 RPM Extract Preparation

Decoction pieces of RPM (1 kg) were refluxed with 10 volumes (10 L) of 70% (v/v) ethanol twice, for 3 h each time. The combined extract was concentrated under reduced pressure, freeze-dried to yield 162.2 g brown powder without residual ethanol and stored in a refrigerator at 4°C until use.

### 2.4 Dosing Solution Preparation

RPM lyophilized powder was prepared at different concentrations of the administration solutions using 0.5% CMC-Na solution. The dosages were 6, 18 and 36 g/kg for the low, middle, and high groups, respectively, which had RPM concentrations of 0.33, 1 and 2 g/ml, respectively. The administration volume was 18 ml/kg.

#### 2.4.1 Pharmacokinetic Study

For the pharmacokinetic (PK) study, three groups (*n* = 6 each) of rats were orally administered RPM extract (6, 18, and 36 g/kg). In the plasma pharmacokinetics experiment, doses were 6, 18, and 36 g/kg for the single administration group and 18 g/kg for the continuous administration group. The doses were set at a subtoxic level (Zhang et al., 2013; Wang et al., 2015) to investigate the pharmacokinetic profiles of the constituents of RPM (36 g/kg group) and were approximately 60 times the upper dose (6 g/day) for humans recommended by the 2020 edition of Chinese Pharmacopoeia (Chinese Pharmacopoeia Commission, 2020), which was converted to a dose for rats based on body surface area conversion (Reagan-Shaw et al., 2008). In addition, the low and medium doses decreased in a certain proportion. Serial blood samples (∼120 μL; before dosing and at 5, 15, and 30 min, and 1, 2, 4, 6, 8, 12, and 24 h after administration) were collected in heparinized tubes from the orbital sinus under light isoflurane. The blood samples were centrifuged at 12000 g for 2 min, and the plasma fractions were decanted and frozen at −70°C until analysis.

#### 2.4.2 Tissue Distribution

For the tissue distribution study, 18 rats were randomly divided into three groups (*n* = 6, each) and orally administered 18 g/kg RPM extract. The rats under isoflurane anesthesia were sacrificed by bleeding from the abdominal aorta at 5, 15, and 60 min (six rats per time point) after gavage with 18 g/kg RPM extract. Subsequently, the heart, liver, spleen, lung, kidney, brain, stomach, small intestine, bladder, and gonads (testes of male rats, and uterus and ovaries of female rats) were immediately collected. An accurately weighed amount of fresh tissue sample (0.25 g) was individually homogenized with normal saline (1 ml) and transferred (50 μL) as a tissue homogenate to 1.5 ml centrifuge tubes for use as the tissue samples. All tissue samples were stored at −70°C until analysis.

#### 2.4.3 Excretion Study

For the excretion study, the rats were housed singly in metabolic cages with urine collection tubes kept at 4 °C during sample collection. Urine and fecal samples were collected at time intervals of 0–4, 4–8, 8–24, 24–32, and 32–48 h after a single dose of RPM extract at 18 g/kg and then weighed. Another six rats were anesthetized with pentobarbital, fixed in the supine position and subjected to bile duct drainage surgery, and the abdominal wound was sutured. Blank bile samples were collected before the administration of RPM. The rats were weighed, and the state of the rats was observed. Subsequently, they were orally administered 18 g/kg RPM extract. The bile samples were collected at time intervals of 0–1, 1–2, 2–4, 4–6, 6–8, 8–24, 24–32 and 32–48 h. The urine, feces, and bile samples were stored at −70°C until use.

### 2.5 Standard Stock Solution and Sample Preparation

Standard stock solutions of chrysophanol, emodin, aloe-emodin, rhein, physcion, questin, citreorosein, questinol, TSG, TG, CG, EG, PG, and IS were prepared in methanol at 1 mg/ml. The calibration samples for the analytes were prepared by adding a series of different concentration working solution solutions (5 μL) to drug-free rat plasma (45 μL). All stock solutions and working solutions were stored at −70°C until use.

Samples were prepared using the protein precipitation process. Rat plasma (50 μL) spiked with 10 μL of IS (puerarin 100 ng/ml) was extracted with 150 μL acetonitrile. After vortex shaking for 5 min and centrifugation at 14,000 g for 5 min, 150 μL of the organic layer was transferred to a clean tube and evaporated to dryness at 25°C under a stream of nitrogen. The residue was reconstituted in 50 μL of the initial mobile phase and subjected to another round of centrifugation at 14,000 g for 5 min, and 5 μL of the supernatant was injected for LC–MS/MS analysis. The tissue samples were homogenized in four parts of ice-cold 0.9% saline solution and then centrifuged at 4°C and 12,000 × g for 10 min to obtain tissue homogenates. Urine and bile samples were centrifuged at 4°C and 12,000 × g for 15 min. Fecal samples were homogenized in 0.9% saline and then centrifuged at 4°C and 12,000 × g for 15 min to gather the fecal homogenates. Tissue homogenate, urine, bile and fecal samples were subjected to a similar process as the rat plasma samples.

### 2.6 LC–MS/MS Instrument and Analytical Conditions

Biological samples were analyzed using UPLC–MS/MS as described in a previous study (Cheng et al., 2020). The analysis was performed on an AB Sciex API 5500 Q Trap mass spectrometer (Toronto, Canada), interfaced with a Waters Acquity UPLC separation module.

Chromatographic separation was achieved on a Waters HSS-T3 C18 column (100 mm × 2.1 mm, 1.8 μm, kept at 40°C) using a mobile phase containing 0.025% formic acid that consisted of solvent A (water) and solvent B (acetonitrile). The mobile phase was delivered at 0.3 ml/min, and the gradient program shown in Supplementary Table S1 was used (Cheng et al., 2020).

The electrospray ionization (ESI) source of the mass spectrometer was operated in negative ion mode. The ion source parameters were optimized as follows: turbo spray temperature, 500°C; nebulizer gas (gas 1), 45 psi; heater gas (gas 2), 50 psi; and curtain gas 45 psi. The dwell time was 50 ms for all analytes. The entrance potential (EP) and collision exit potential (CXP) were set at –10 and –16 V, respectively. The declustering potential (DP), collision energy (CE) and other detailed mass spectrometry conditions were applied according to Cheng et al. (2020).

### 2.7 Data Analysis

The pharmacokinetic parameters, including the area under the plasma concentration-time curve from time zero to time t (AUC_0-t_) to time infinity (AUC_0-∞_), the time to reach the maximum plasma concentration (*T*_max_), the elimination half-time (*t*_1/2_), and the maximum plasma concentration (*C*_max_), were calculated using the pharmacokinetic software Phoenix^®^ WinNonlin^®^, version 7.0 (Scientific Consulting Inc., Apex, NC, United States). Data are expressed as the mean ± standard deviation (SD) for each group. The dose-exposure relationship was evaluated by the power function model combined with the confidence interval method (Smith et al., 2000; Sethuraman et al., 2007; Hummel et al., 2009). First, the administration dose, AUC_0-∞_ and *C*_max_ were logarithmically transformed, and then a linear regression was performed to obtain the curve correlation coefficient (R^2^), slope and 90% confidence interval (90% CI).

## 3 Results and Discussion

The LC–MS/MS method for the assay of 13 components from RPM in multiple biological samples was validated based on the Bioanalytical Method Validation Guidance for Industry issued by the Center for Drug Evaluation and Research and the Center for Veterinary Medicine of the US Food and Drug Administration (U.S. Department of Health and Human Services, Food and Drug Administration, Center for Drug Evaluation and Research, Center for Veterinary Medicine, 2018). For the method of validating rat plasma, see previously published articles (Cheng et al., 2020). The bioanalytical methods for validating rat tissue, bile, urine and feces are shown in Supplementary Figures S1–S4 and Supplementary Tables S2–S5. After validation, the method was successfully applied to determine the pharmacokinetics, tissue distribution, and excretion characteristics study in rats following oral administration of the RPM extract.

### 3.1 Pharmacokinetics of the RPM Extract After Oral Administration in Rats

Previous studies reporting the pharmacokinetics of RPM have mainly focused on a single component. However, the constituents of RPM are complex, and there may be interactions and mutual transformations between them. Therefore, monomers can be used to study medicinal materials, and obvious differences will be observed in the overall pharmacokinetic studies. In this experiment, UPLC–MS/MS was used to analyze the pharmacokinetics of 13 active ingredients in the RPM extract orally administered at three different doses. The calculated doses of the components based on the contents in the extract were 7.38, 14.8, 13.7, 8.82, 15.5, 11.2, 55.8, 1.08, 1,170, 11.3, 25.7, 131.4, and 13.0 mg/kg for chrysophanol, emodin, aloe-emodin, rhein, physcion, questin, citreorosein, questinol, TSG, TG, CG, EG, and PG, respectively.

Increasing dosage is a useful way to facilitate uncovering the herbal compounds (unchanged and/or metabolized forms) with significant levels in general circulation and in target tissue. Therefore, we hope to set the dosage of the high-dose group as high enough to fully reveal the exposure of the target compounds in vivo in this study. However, after 28 days of continuous gavage of 40 g/kg ethanol extract of Polygonum multiflorum, the liver, kidney and lung of rats were damaged (Qi et al., 2013). In consideration of the welfare of experimental animals and the convenience of experimental operation, the concentration of the extract solution administered to rats should not be too high, and the administration volume should not be too large. In this way, the dose of 36 g/kg RPM extract was finally set as the high dose level with the concentration and volume of the administration solution 2 g/mL and 18 mL/kg, respectively, in this study.

**FIGURE 2:**
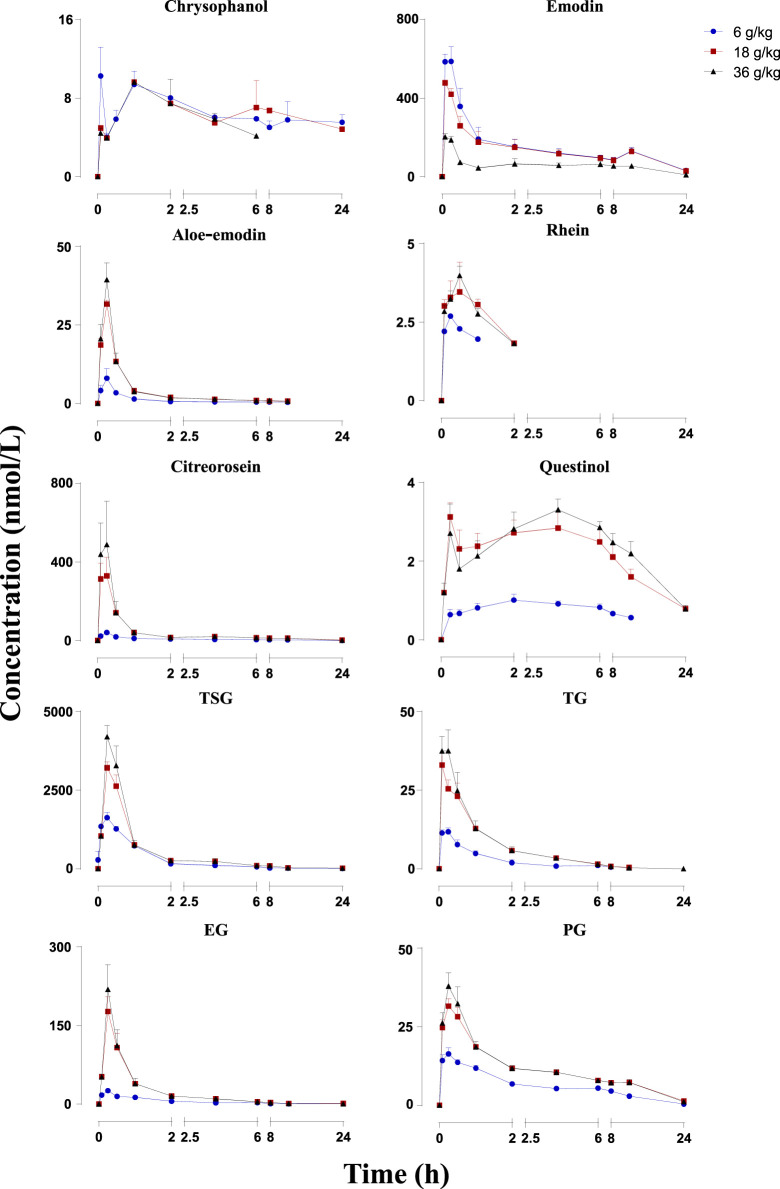
Mean plasma concentration-time profiles of the 10 constituents after administration of RPM (6, 18 and 36 g/kg) to rats. The upper error bars represent the standard deviation obtained from six replicates.

The mean plasma concentration–time profiles are illustrated in Figure 2, and the pharmacokinetic parameters are presented in Table 1. The plasma concentrations of physcion, questin, and CG were too low to be detected, whereas rhein could not be detected in rats dosed with 6 g/kg RPM. Physcion has a moderate intestinal permeability with P_eff_ values of (3.32 ± 1.50) × 10^–3^ (2.30 ± 1.57) × 10^–3^ (2.40 ± 0.58) × 10^–3^, and (7.45 ± 3.30) × 10^–3^ cm/min in the duodenum, jejunum, ileum, and colon, respectively (Wang P. et al., 2011). Rhein is mostly administered orally due to its low solubility, although achieving a stable and effective blood concentration is difficult (Li et al., 2021). The components detected in the plasma gradually increased as the dose increased. Seven constituents detected in this study, namely, emodin, aloe-emodin, citreorosein, TSG, TG, EG and PG, exhibited *C*_max_ values within 20 min, thus demonstrating rapid gastrointestinal tract absorption. TSG had the highest *C*_max_ due to its good water solubility and a log*P* value of 0.10, and it content was highest among the 13 constituents in the RPM extract. Although emodin has poor water solubility with a high log*P* (5.03), it reached the second highest *C*_max_ due to its high content in the extract and fast absorption (Liu et al., 2010). The *C*_max_ values of the other ingredients, namely, chrysophanol, rhein, aloe-emodin, citreorosein, questinol, TG, EG, and PG, were relatively low. The low *C*_max_ of chrysophanol and rhein may be due to their low content in the RPM extract. Aloe-emodin is rapidly absorbed and quickly metabolized to rhein and other unknown metabolites in the body (Lang, 1993). In the Caco-2 cell model and the everted gut sac model, aloe-emodin was mainly absorbed in a glucuronidated or sulfated form, suggesting that a significant amount was transformed during absorption (Park et al., 2009). The index AUC_0-∞_, which reflects the degree of exposure to the drug in the body, also showed the same trend (Supplementary Figure S6), with the highest exposure observed for TSG and then emodin in a dose-dependent manner.

**TABLE 1:** Pharmacokinetics of the 10 constituents in rats after p.o. administration of RPM extract at dose of 6, 18, and 36 g/kg (Mean ± SD, *n* = 6).

Compound	Chrysophanol			Emodin			Aloe-emodin		
Dosing	6 g/kg	18 g/kg	36 g/kg^a^	6 g/kg	18 g/kg	36 g/kg^a^	6 g/kg	18 g/kg	36 g/kg^a^
*C*_max_ (nmol/L)	9.36 ± 3.26	7.87 ± 2.19	7.48 ± 2.21	205 ± 39.4	491 ± 35.8	648 ± 125	8.25 ± 7.47	31.7 ± 3.03	42.0 ± 11.5
*T*_max_(h)	1.03 ± 0.96	3.51 ± 3.06	2.22 ± 1.76	0.12 ± 0.07	0.14 ± 0.09	0.19 ± 0.09	0.29 ± 0.10	0.25	0.22 ± 0.07
*t*_1/2_ (h)	26.3 ± 14.4	–	3.18 ± 0.62	7.72 ± 3.67	9.44 ± 3.63	8.37 ± 4.17	8.46 ± 7.14	5.09 ± 3.38	3.44 ± 1.40
AUC_0-t_ (nmol·h/L)	74.8 ± 58.9	19.8 ± 9.41	16.9 ± 6.47	1,040 ± 133	2,480 ± 558	2,540 ± 694	8.32 ± 4.16	25.4 ± 9.70	27.0 ± 12.0
AUC_0-∞_ (nmol·h/L)	340 ± 195	–	40.4 ± 7.36	1,350 ± 504	2,910 ± 707	2,970 ± 863	12.4 ± 7.0	32.4 ± 14.4	31.3 ± 12.7
MRT_0-∞_ (h)	40.3 ± 23.3	–	6.76 ± 2.06	12.7 ± 4.65	13.0 ± 4.18	11.8 ± 5.27	9.61 ± 8.77	4.65 ± 3.53	3.17 ± 1.54

**Compound**	**Rhein**			**Citreorosein**			**Questinol**		
**Dosing**	**6 g/kg**	**18 g/kg**	**36 g/kg^a^**	**6 g/kg**	**18 g/kg**	**36 g/kg^a^**	**6 g/kg**	**18 g/kg**	**36 g/kg^a^**
*C*_max_ (nmol/L)	–	3.72 ± 1.25	3.74 ± 0.84	41.2 ± 8.80	349 ± 216	521 ± 512	1.09 ± 0.317	3.54 ± 0.652	4.00 ± 0.673
*T*_max_(h)	–	0.42 ± 0.14	0.46 ± 0.10	0.25	0.19 ± 0.09	0.19 ± 0.09	3.50 ± 2.17	1.83 ± 1.80	4.38 ± 4.03
*t*_1/2_ (h)	–	–	1.18 ± 0.39	5.07 ± 1.32	3.78 ± 0.75	3.97 ± 1.31	8.00 ± 2.01	14.4 ± 9.99	8.90 ± 2.70
AUC_0-t_ (nmol·h/L)	–	4.26 ± 2.99	3.91 ± 2.23	87.2 ± 21.5	416 ± 251	458 ± 338	8.61 ± 1.69	40.1 ± 8.98	47.3 ± 6.85
AUC_0-∞_ (nmol·h/L)	–	–	8.14 ± 1.78	96.1 ± 17.1	558 ± 499	468 ± 337	27.0 ± 22.8	58.3 ± 14.3	57.5 ± 9.45
MRT_0-∞_ (h)	–	–	1.83 ± 0.56	6.87 ± 1.42	5.69 ± 1.57	5.32 ± 1.85	12.2 ± 2.93	20.6 ± 13.5	13.6 ± 4.21

Compound	TSG			TG		
Dosing	6 g/kg	18 g/kg	36 g/kg^a^	6 g/kg	18 g/kg	36 g/kg^a^
*C*_max_ (nmol/L)	1,670 ± 403	3,260 ± 494	4,290 ± 986	12.4 ± 2.66	37.3 ± 4.57	45.8 ± 9.94
*T*_max_(h)	0.24 ± 0.15	0.33 ± 0.13	0.33 ± 0.13	0.19 ± 0.09	0.11 ± 0.07	0.11 ± 0.07
*t*_1/2_ (h)	6.53 ± 2.45	6.54 ± 2.02	5.98 ± 2.62	1.23 ± 0.58	1.90 ± 0.22	2.00 ± 0.63
AUC_0-t_ (nmol·h/L)	2,380 ± 280	4,040 ± 1,050	4,460 ± 1,360	15.8 ± 10.6	48.0 ± 17.2	51.9 ± 18.1
AUC_0-∞_ (nmol·h/L)	2,460 ± 273	4,200 ± 1,080	4,610 ± 1,430	16.6 ± 10.9	49.5 ± 17.1	53.0 ± 18.3
MRT_0-∞_ (h)	3.86 ± 1.17	4.37 ± 0.91	3.66 ± 0.97	1.58 ± 0.77	2.23 ± 0.30	2.10 ± 0.24

Compound	EG			PG		
Dosing	6 g/kg	18 g/kg	36 g/kg^a^	6 g/kg	18 g/kg	36 g/kg^a^
*C*_max_ (nmol/L)	25.7 ± 5.73	187 ± 68.5	233 ± 110	16.8 ± 4.10	34.2 ± 4.59	42.1 ± 10.9
*T*_max_(h)	0.25	0.29 ± 0.10	0.29 ± 0.10	0.29 ± 0.10	0.26 ± 0.13	0.26 ± 0.13
*t*_1/2_ (h)	3.54 ± 1.55	4.85 ± 1.99	3.92 ± 2.50	4.83 ± 0.99	7.09 ± 2.12	6.13 ± 1.06
AUC_0-t_ (nmol·h/L)	46.7 ± 24.6	186 ± 73.4	191 ± 74.3	77.7 ± 6.12	176 ± 16.8	178 ± 20.1
AUC_0-∞_ (nmol·h/L)	48.8 ± 24.4	194 ± 79.9	194 ± 74.7	91.9 ± 14.5	192 ± 12.6	188 ± 18.6
MRT_0-∞_ (h)	4.40 ± 1.79	3.87 ± 1.87	2.88 ± 1.41	7.55 ± 1.48	9.64 ± 2.05	8.58 ± 1.72

a The pharmacokinetic parameters of the 36 g/kg dose have been published in Cheng et al., 2020.

Subsequently, we performed a regression analysis using the log-transformed values of the dose, AUC_0-∞_ and *C*_max_ (Supplementary Figure S6, Tables 2, 3). The AUC_0-∞_ and *C*_max_ of the active ingredient of the RPM extract were directly proportional to the dose except for rhein and chrysophanol. The AUC_0-∞_ of chrysophanol, emodin, questinol, TSG and PG were nonlinearly related to the dose (90% CI of the slope were beyond the critical interval of 0.88–1.12), whereas the AUC_0-∞_ and dose of aloe-emodin, citreorosein, TG, and EG showed an uncertain linear relationship (90% CI of the slope of some samples were within the critical interval of 0.88–1.12). Additionally, the *C*_max_ of emodin, TSG, TG, and PG was nonlinearly related to the dose (90% CI of the slope was beyond the critical interval of 0.80–1.20), whereas those of aloe-emodin, citreorosein, questinol, and EG could not be linearly correlated with the dose (90% CI of the slope of some samples was within the critical interval of 0.80–1.20). Understanding the pharmacokinetics of a drug, which affects drug availability at the target site is essential to eliciting the desired effects of a drug on its target.

**TABLE 2:** Relationships between system exposure level (AUC_0-∞_) of the 10 constituents and p.o. dose of RPM extract in rats.

Compound	R^2^	*P*	Slope (90%CI)	Conclusion
Chrysophanol	0.288	2.63 × 10^–2^	–0.667 (–1.14 to –0.193)	Nonlinear correlation
Emodin	0.573	2.77 × 10^–4^	0.466 (0.290–0.642)	Nonlinear correlation
Aloe-emodin	0.430	4.29 × 10^–3^	0.582 (0.278–0.885)	Uncertainty of linear relationship
Citreorosein	0.448	2.37 × 10^–3^	0.818 (0.422–1.21)	Uncertainty of linear relationship
Questinol	0.491	3.64 × 10^–3^	0.551 (0.275–0.827)	Nonlinear correlation
TSG	0.539	5.20 × 10^–4^	0.342 (0.204–0.479)	Nonlinear correlation
TG	0.600	1.61 × 10^–4^	0.720 (0.464–0.977)	Uncertainty of linear relationship
EG	0.592	1.90 × 10^–4^	0.834 (0.520–1.11)	Uncertainty of linear relationship
PG	0.767	1.89 × 10^–6^	0.429 (0.326–0.532)	Nonlinear correlation

**TABLE 3:** Relationship between system exposure level (*C*_max_) of the 10 constituents and p.o. dose of RPM extract in rats.

Compound	R^2^	*P*	Slope (90%CI)	Conclusion
Chrysophanol	0.147	0.142	–0.158 (-0.337–0.021)	Nonlinear correlation
Emodin	0.887	5.37 × 10^–9^	0.656 (0.554–0.758)	Nonlinear correlation
Aloe-emodin	0.740	4.73 × 10^–6^	1.07 (0.795–1.35)	Uncertainty of linear relationship
Rhein	0.002	0.904	0.034 (-0.482–0.550)	Nonlinear correlation
Citreorosein	0.644	6.08 × 10^–5^	1.27 (0.857–1.68)	Uncertainty of linear relationship
Questinol	0.810	3.71 × 10^–7^	0.774 (0.610–0.938)	Uncertainty of linear relationship
TSG	0.810	3.67 × 10^–7^	0.536 (0.422–0.649)	Nonlinear correlation
TG	0.868	1.91 × 10^–8^	0.756 (0.627–0.884)	Nonlinear correlation
EG	0.766	2.02 × 10^–6^	1.23 (0.931–1.52)	Uncertainty of linear relationship
PG	0.762	2.25 × 10^–6^	0.524 (0.396–0.652)	Nonlinear correlation

For enhanced effects, drugs must enter the systemic circulation and reach the target site of action to interact with receptors. Therefore, pharmacokinetic analyses to determine a drug’s significant systemic exposure after administration have gained increasing research interest (Li, 2017). This study showed that after the oral administration of RPM extract to SD rats, TSG, which has with a shorter half-life and faster elimination speed was absorbed into the blood in the form of a prototype component in the stomach. These observations are consistent with those reported in previous studies (Sun et al., 2005). In addition, P-glycoprotein and multidrug resistance-associated protein two are also involved in the absorption of TSG in the intestine (Lei et al., 2015; Liu et al., 2019; Wang et al., 2020). The absorption of emodin was also better, and its main absorption sites are the duodenum and jejunum (Liu et al., 2010). After emodin intragastric administration, emodin rapidly undergoes phase II metabolism to form its glucuronide. In addition, transporters, including Na^+^/glucose cotransporter (SGLT1), MRP2, and P-glycoprotein, are also involved in the efflux transport of emodin (Liu et al., 2012). The exposure to emodin in RPM extract was also high, and the exposure level also gradually increased with an increase in the dose. TSG could further increase the hepatotoxicity of emodin by enhancing the absorption of emodin and inhibiting its metabolism (Li et al., 2020). Consistent with the findings of this study, a previous study comparing the absorption of various anthraquinone components using a Caco-2 cell model demonstrated that the absorption of chrysophanol was the lowest (Wang and yang, 2008). Citreorosein and questinol ether are rarely reported; however, citreorosein presented a higher exposure with rapid and better absorption and faster elimination. In contrast, questinol ether presented a lower exposure level, poor absorption, and slower elimination. These results suggest that citreorosein could have a higher research value than questinol ether. These components are combination anthraquinones. Although several reports are available on EG, few studies have explored the pharmacokinetics of TG and PG. Here, we show that the exposure levels of the latter two in the body were not low and increased with an increase in the administered dose, and they also showed an accumulation effect. The cause of liver toxicity of Polygonum multiflorum is related to anthraquinones (Ruili et al., 2019). The possible mechanisms of anthraquinone-induced hepatotoxicity include inducement of lipid peroxidation in hepatocytes (Guoxin et al., 2019); inhibition of hepatocyte growth, which leads to hepatocyte apoptosis (Ronghua et al., 2016); and changes in the function and expression of bile acid transporters and metabolic enzymes, abnormal structure and metabolic function of liver cells (Pelkonen et al., 2015), and leads to oxidative stress damage of liver cells (Lei et al., 2015). Emodin, aloe emodin, chrysophanol and rhein exhibit cytotoxicity to normal human hepatocytes HepaRG and cause damage to hepatocyte DNA to different degrees (Lei et al., 2015). Research has shown that the hepatotoxic effect of these components on LO2 human hepatocytes was investigated, and EG, PG, emodin and physcion were finally confirmed to be at least partial hepatotoxic components (Lv et al., 2015). In this experiment, emodin and EG exposure was higher. Although citreorosein is less reported, it is also presents high exposure *in vivo*. These compounds easily accumulate *in vivo* with prolonged administration time, and they which may be the main factors that to induce hepatocellular toxicity; thus, they and deserve further study.

These findings revealed the exposure level, absorption, and elimination rate of the active components of RPM in rats. Of the several investigated components, TSG, emodin, TG and PG showed the most obvious results.

### 3.2 Tissue Distribution

**FIGURE 3:**
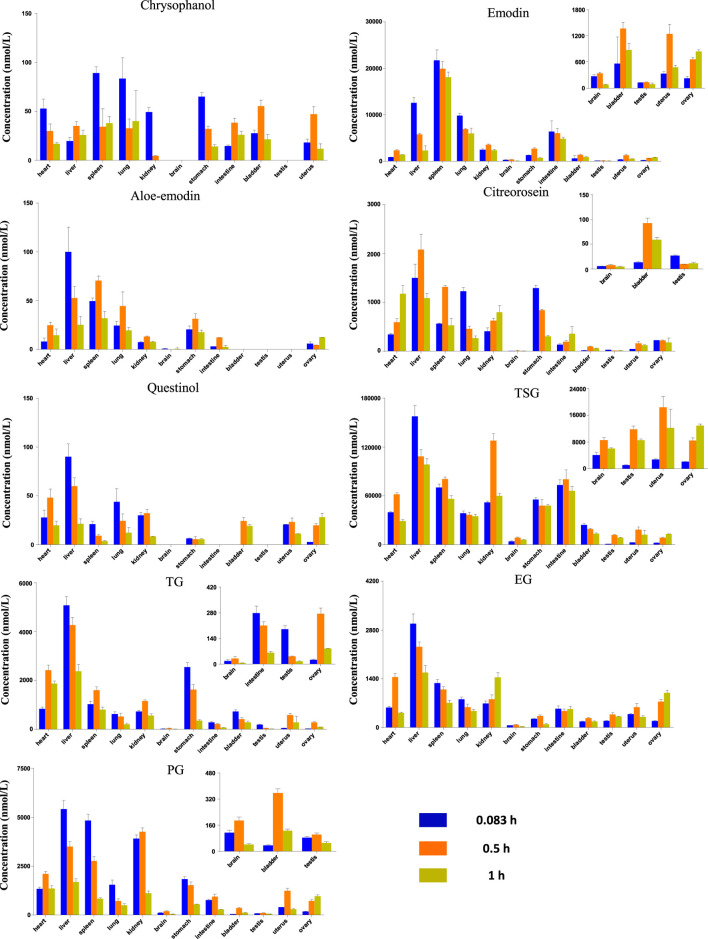
Tissue distribution of the RPM constituents in different sampling time after rat orally administration of RPM extract (18 g/kg).

The study of tissue distribution is one of the important areas of pharmacokinetics research because such distributions are closely related to the pharmacology, toxicity, and side effects of a drug. A drug must present pharmacological activity and effective concentrations of its chemical components at the target site to produce pharmacodynamic effects on the body. Therefore, the level of exposure to a compound in the tissue directly impacts its efficacy. The results showed that after 5, 15 and 60 min of oral administration of the RPM extract, each compound was distributed over various tissues throughout the body, suggesting that the body absorbs each active substance rapidly (Figure 3). Moreover, the distributions of TSG, emodin, and PG were relatively higher in each tissue compared with the other compounds (Supplementary Table S11), which could be related to their higher contents in the extract.

Drugs are distributed throughout various body tissues by systemic circulation after absorption into the blood, regardless of the administration method. Drugs enter tissues that receive high blood flow first, followed by those that receive low blood flow. Our study demonstrated that the nine key compounds in RPM were mainly distributed in the liver and kidney but presented a limited distribution in the bladder and gonads (Supplementary Table S11).

In addition to receiving higher blood flow, the liver is also an important metabolic organ, and drugs absorbed through the digestive tract enter the liver through the hepatic portal vein and are converted into other products under the action of liver drug enzymes. Modern pharmacological studies have shown that RPM can reduce hepatomegaly caused by CCL4, inhibit liver microsomal lipid peroxidation caused by ADP and ADP-reduced coenzymes, and reduce serum free fatty acid and lipid peroxide levels (Li et al., 2017). The liver injury caused by RPM was mostly hepatocellular injury, followed by mixed liver injury and cholestatic liver injury. Although RPM can cause varying degrees of liver injury and even death, most RPM-related liver injuries are reversible after stopping RPM products and conservative treatment (Zhu et al., 2015). Some *in vitro* studies have shown that anthraquinones from RPM, including emodin, chrysophanol, and physcion, can change the distribution of bile acids in sandwich-cultured rat hepatocytes (Kang et al., 2017). However, emodin in RPM can induce the expression of cytochrome P450 enzyme mRNA and damage normal human liver cells (Wang et al., 2016). Taken together, these results indicate that the liver is the target organ of RPM’s medicinal and toxic effects. Furthermore, studies have shown that TSG, an effective RPM component, can inhibit the NO-ONOO renal oxidative stress pathway in early diabetic nephropathy and reduce the production of nitrotyrosine, thereby inhibiting kidney function damage caused by early oxidative stress in diabetic nephropathy (Yuan and Liu, 2016). In contrast, another study showed that emodin and rhein could significantly inhibit the proliferation of HK-2 cells through the MAPK/ERK signaling pathway to inhibit the phosphorylation of ERK and change the proportion of its lipid part, which leads to damage to the outer mitochondrial membrane of renal tubular epithelial cells (Wang et al., 2007), thus indicating that the kidney is also a prime target organ for the medicinal and toxic effects of RPM. Moreover, experimental studies have shown that the exposure of TSG in various tissues is higher than that of other compounds with a higher distribution in brain tissues (Supplementary Table S11), and pharmacokinetic studies have shown that TSG plays a role in improving learning and memory abilities (Wang T. et al., 2011). In addition to TSG, emodin, EG, and PG are also distributed in small amounts in the brain. Therefore, it would be worthwhile to study the pharmacological activities of these compounds on the central nervous system.

Compared with other organs, emodin and chrysophanol had the highest exposure in the spleen (AUC_0-t_ of 19,100 and 50.3 nmol h/L, respectively), whereas the remaining seven compounds had the highest exposure in the liver tissue (Supplementary Table S11). Chrysophanol, aloe-emodin, and questinol ether were rarely exposed in the liver, which could be related to their lower content in the extract. The AUC_0-t_ of TSG in the kidney was 86,400 nmol h/L, and the AUC_0-t_ of the other compounds varied from 9.77 to 3,210 nmol h/L. In addition, chrysophanol, aloe-emodin, and questinol ether were rarely exposed in the kidneys, which was also related to their lower content in the extract.

Preliminary studies have shown that the exposure of the active ingredients of RPM in the body is positively correlated with the RPM dose, and the current study further indicates that most of the active components of RPM are distributed in liver and kidney tissues. In addition, among the active components of RPM, emodin, TSG, and EG are exposed to higher levels and can cause liver or kidney damage (Wu et al., 2018), suggesting that long-term exposure to higher doses of RPM extract could cause the highest damage to the liver and kidney.

Collectively, these findings demonstrated that after orally administering RPM extract to rats, its active ingredients can be rapidly distributed to several tissues and organs, among which the liver and kidney are the main organs. In addition to TSG, emodin, EG and PG are distributed in the brain. The study also shows that the active ingredients of RPM are eliminated quickly from tissues.

### 3.3 Excretion Into Bile, Urine and Feces

**FIGURE 4:**
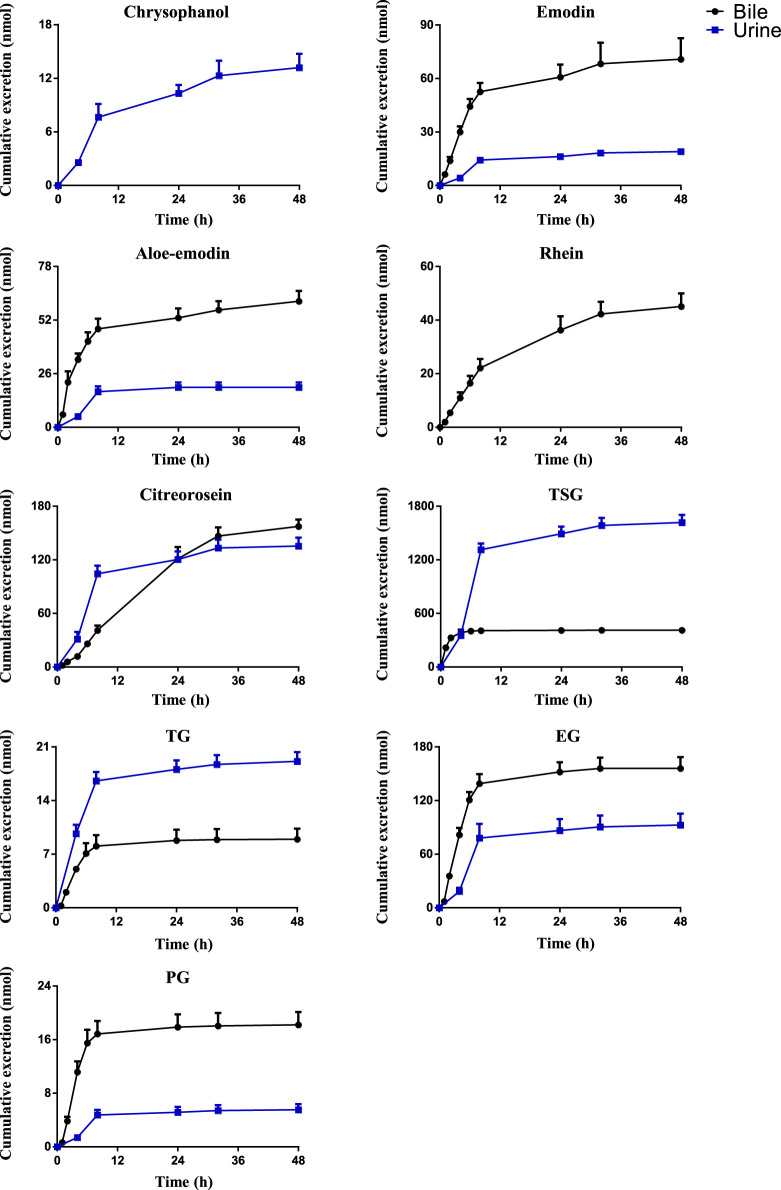
The mean cumulative urinary and biliary excretion profile of the eight constituents in rat after single p.o. administration of RPM extract at 18 g/kg. The upper error bars represent the standard deviation obtained from six replicates.

**FIGURE 5:**
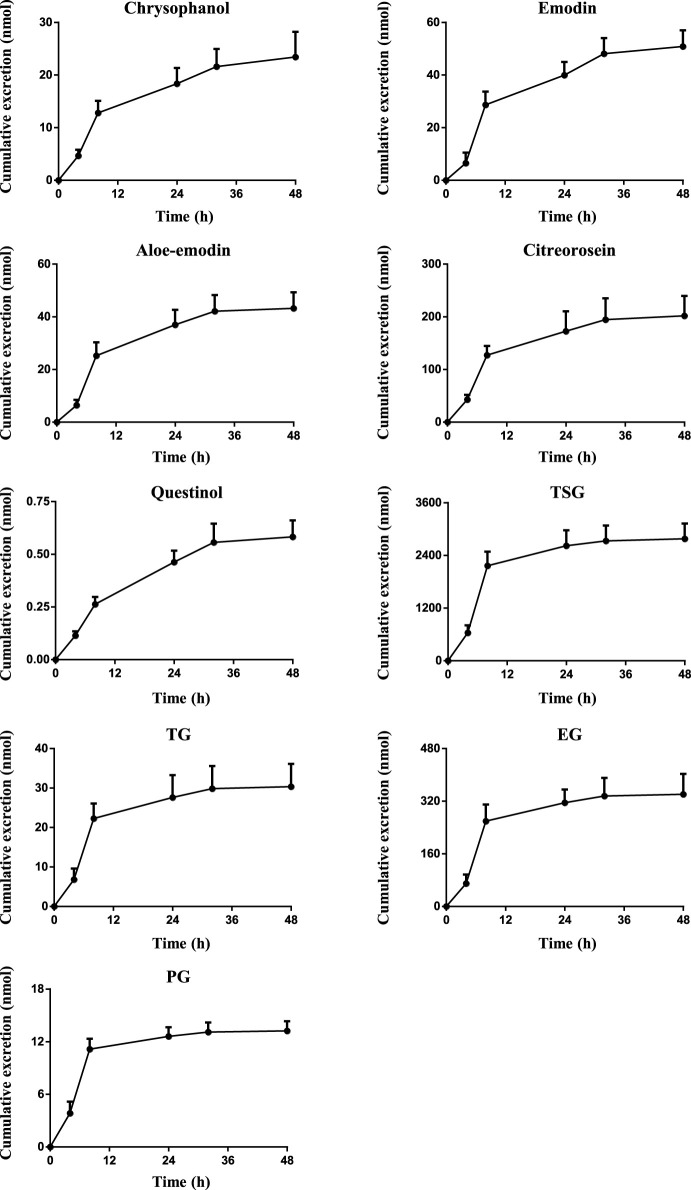
The mean cumulative fecal excretion profile of the eight constituents in rat after single p.o. administration of RPM extract at 18 g/kg. The upper error bars represent the standard deviation obtained from six replicates.

Drugs and their metabolites are excreted from the body through excretory processes, such as through renal, bile, and intestinal excretions. With the continuous in-depth study of pharmacokinetics, research on drug excretion kinetics has also received increased attention. However, few reports have focused on the excretion kinetics of RPM extract. Therefore, this study determined the contents of the active ingredients of RPM extract in rat urine, feces, and bile using the UPLC–MS/MS method. Combined with a study of the plasma pharmacodynamics and tissue distribution, the excretion of multiple components of RPM in rats was analyzed (Supplementary Table S7, Figures 4, 5). Although questinol and PG presented low recovery from the urine and feces at 4.12% and 16.40%, respectively, the remaining compounds, namely, chrysophanol, emodin, aloe-emodin, citreorosein, questinol, TSG, TG and EG, presented higher recovery from the urine and feces at 28.67%–45.50% (see Supplementary Table S8 for specific data). The results demonstrated that most of the compounds could be detected in urine, feces, and bile after oral administration of RPM extract, with higher values observed in the fecal samples than the urine samples. This finding could be because the components in feces are obtained from several sources such as unabsorbed drug components, components secreted by bile into the intestinal lumen, resecretion in the intestines re-secretion, and metabolic transformations by gut flora.

The results showed that the excretion of citreorosein, TSG, TG, and EG in feces was higher than that in urine and bile. Tissue distribution studies have shown that citreorosein presents a greater distribution in the liver while its secretion from bile was 158 ± 13.2 nmol, indicating that this component is mainly metabolized by the liver. The average cumulative excretion of the components in feces increased slowly, even after 32 h of administration, which suggests that the prototype constituent in the feces is primarily associated with bile secretion or combined anthraquinone decomposition in the intestine and liver. Moreover, the tissue distribution study revealed a higher distribution of TSG in gastrointestinal tissues than the liver, and the amount excreted by bile secretion was far less than the cumulative amount excreted in feces. These results suggested that the high content of components in feces could be related to the excretion of components through the intestinal epithelium. TSG undergoes phase II metabolism in the liver, a few components are excreted through bile, and most components are excreted in feces (Wang et al., 2009). Additionally, consistent with the higher distribution of TSG in the kidneys the accumulated urine excretion was also higher, indicating that urine excretion is another key excretion method of TSG. In contrast, the cumulative bile excretion of TG and EG was much lower than the cumulative excretion in feces despite these components showing a relatively higher distribution in the liver than the kidney, indicating that the higher content of these components in feces could have been contributed by bile secretion through the intestinal epithelium. PG is mainly excreted by bile, but its cumulative excretion in feces was lower than that in bile, suggesting that PG might have entered the hepatoenteric circulation. Moreover, its excretion in urine was lower despite its higher distribution in the kidneys than in the other organs, suggesting that the compound could have an accumulation effect in the kidneys, thereby causing the nephrotoxicity of RPM. The excretion rate of emodin in bile was higher than that in urine but did not differ remarkably from that in feces. Emodin is mainly metabolized in the liver, which could be the reason for the high excretion rate of emodin in bile. However, the average cumulative excretion-time curve of the three excretion pathways revealed a slowly increasing trend of emodin, even after 32 h of administration, which could be due to a longer residence time of the compound in rats and its high content in various tissues. Rhein was mainly excreted by bile, whereas it was not detected in urine or feces, suggesting that rhein might have entered the hepato-intestinal circulation, which led to rendering its further metabolization and transformation after reabsorption through the intestine. However, there was no double peak in the blood concentration-time curve, which indicates that the component might have been converted into other products in the gastrointestinal tract and absorbed or excreted by the body. Furthermore, the average cumulative excretion-time curve of chrysophanol and aloe-emodin slowly increased after administration, which may have been related to the slow elimination of each compound, the tissue hydrolysis of other compounds in the gastrointestinal tract or transformation by hepatic drug enzyme metabolism.

Chrysophanol and questinol ether were not detected in bile. Questinol ether may not have been detected in urine and bile because of its low content in the RPM extract, low absorption in the body, or conversion into other products to be excreted. The absence of chrysophanol in bile, but its presence in feces could be related to the low bioavailability of this component or the secretion of intestinal epithelial cells. Its absence in bile indicates that liver drug enzymes could have converted the component into other products with a faster conversion rate or that bile does not excrete the component.

In summary, most of the active ingredients of RPM can be excreted through urine excretion, bile secretion and intestinal epithelial cell secretion. Among them, PG, rhein, and emodin showed the possibility of entering the hepatointestinal circulation, wherein TSG, chrysophanol, TG, and EG are mainly excreted by intestinal epithelial cells and urine.

## 4 Conclusion

In conclusion, after oral administration of RPM extracts to SD rats, the active ingredients were quickly absorbed, including emodin, TSG, citreorosein, TG, EG and PG. These components were exposed at high levels in the body, and revealed a certain accumulation effect with increasing doses; thus, they are worthy of further in-depth study. Furthermore, the distributions of emodin, TSG, TG, EG, and PG in the liver and kidney were high, indicating that these components could be the main substances with pharmacodynamic and toxic effects on the liver and kidney. The excretion pathway of each active ingredient followed mainly fecal and bile excretion, whereas several were converted into other products and excreted through the urine and feces. Herbal medicines are complex mixturesthat may interact with a wide range of proteins in various metabolic organs in extremely complex ways (Ge, 2019; Liu W. et al., 2021). The interaction between drugs and herbs has received increasing attention. The interaction between the components of RPM will also become the focus of future research. Collectively, the results of this study would be helpful for developing new medicines related to RPM and identifying the safety of these medicines by providing insights on the pharmacokinetic characteristics of RPM.

## Data Availability

The original contributions presented in the study are included in the article/Supplementary Material, further inquiries can be directed to the corresponding authors.

## Abbreviations

CE, collision energy; CG, chrysophanol-8-O-β-D-glucoside; CMC-Na, carboxymethyl cellulose; CXP, collision exit potential; DP, declustering potential; EG, emodin-8-O-β-D-glucoside; EP, entrance potential; ESI, electrospray ionization; IS, internal standard; PG, physcion-8-O-β-D-glucoside; PK, pharmacokinetic; RPM, radix polygoni multiflori; TG, torachrysone-8-O-glucoside; TSG, 2,3,5,4′-teterahydroxystilbene-2-O-β-D-glucoside; UPLC-MS/MS, ultra-performance liquid chromatography-tandem mass spectrometry.

## References

[B1] Ahn S. M. Kim H. N. Kim Y. R. Choi Y. W. Kim C. M. Shin H. K. (2016). Emodin from Polygonum Multiflorum Ameliorates Oxidative Toxicity in HT22 Cells and Deficits in Photothrombotic Ischemia. J. Ethnopharmacol. 188, 13–20. 10.1016/j.jep.2016.04.058 27151150

[B2] Büchter C. Zhao L. Havermann S. Honnen S. Fritz G. Proksch P. (2015). TSG (2,3,5,4'-Tetrahydroxystilbene-2-O- β -D-Glucoside) from the Chinese Herb Polygonum Multiflorum Increases Life Span and Stress Resistance of *Caenorhabditis elegans* . Oxid. Med. Cel. Longev. 2015, 124357. 10.1155/2015/124357 PMC4436517 26075030

[B3] Chang Y. X. Ge A. H. Jiang Y. Teye Azietaku J. Li J. Gao X. M. (2016). A Bioactivity-Based Method for Screening, Identification of Lipase Inhibitors, and Clarifying the Effects of Processing Time on Lipase Inhibitory Activity of Polygonum Multiflorum. Evid. Based Complement. Alternat Med. 2016, 5965067. 10.1155/2016/5965067 26925151 PMC4746387

[B4] Chen Q. Zhang S.-z. Ying H.-z. Dai X.-y. Li X.-x. Yu C.-h. (2012). Chemical Characterization and Immunostimulatory Effects of a Polysaccharide from Polygoni Multiflori Radix Praeparata in Cyclophosphamide-Induced Anemic Mice. Carbohydr. Polym. 88 (4), 1476–1482. 10.1016/j.carbpol.2012.02.055

[B5] Cheng W. H. Ye H.-c. Yang W. Wu S. Y. Wei M. M. Gao Y. (2020). Simultaneous Determination of 13 Constituents of Radix Polygoni Multiflori in Rat Plasma and its Application in a Pharmacokinetic Study. Int. J. Anal. Chem. 2020, 1–10. 10.1155/2020/4508374 PMC7072103 32190053

[B6] Cheung F. W. Leung A. W. Liu W. K. Che C. T. (2014). Tyrosinase Inhibitory Activity of a Glucosylated Hydroxystilbene in Mouse Melan-A Melanocytes. J. Nat. Prod. 77 (6), 1270–1274. 10.1021/np4008798 24933607 PMC4076036

[B7] Chinese Pharmacopoeia Commission (2020). Pharmacopoeia of the People’s Republic of China. Beijing, China: China Medical Science Press.

[B8] Choi S. G. Kim J. Sung N. D. Son K. H. Cheon H. G. Kim K. R. (2007). Anthraquinones, Cdc25B Phosphatase Inhibitors, Isolated from the Roots of Polygonum Multiflorum Thunb. Nat. Prod. Res. 21 (6), 487–493. 10.1080/14786410601012265 17497420

[B9] En-Ze L. I. Yuan Y. Shao H. Pharmacology D. O. (2016). Study of the Analytical Method of Rhein and its Activated Metabolites in Liver Microsome Incubations. J. Southeast Univ. (Med. ence Ed.) 35 (6), 894–899. 10.3969/j.issn.1671-6264.2016.06.013

[B10] Fan W. Guo Y. Cao S. Cao S. Xie Y. Liu X. (2021). Tetrahydroxystilbene Glucoside Alleviates Angiotensin II Induced HUVEC Senescence via SIRT1. Can. J. Physiol. Pharmacol. 99 (4), 389–394. 10.1139/cjpp-2020-0202 32898442

[B75] Ge G. B. (2019). Deciphering the Metabolic Fates of Herbal Constituents and the Interactions of Herbs with Human Metabolic System. Chin. J. Nat. Med. 17 (11), 801–802. 10.1016/S1875-5364(19)30098-6 31831127

[B12] Guoxin L. Zinan L. Junyu N. (2019). General Situation of Studies on Anthraquinone Toxicity and its Mechanism. Jounral Toxicol. 33 (1), 70–74. 10.16421/j.cnki.1002-3127.2019.01.018

[B13] Hou H. Li D. Cheng D. Li L. Liu Y. Zhou Y. (2013). Cellular Redox Status Regulates Emodin-Induced Radiosensitization of Nasopharyngeal Carcinoma Cells *In Vitro* and *In Vivo* . J. Pharm. (Cairo) 2013, 218297. 10.1155/2013/218297 26555969 PMC4590808

[B14] Hummel J. McKendrick S. Brindley C. French R. (2009). Exploratory Assessment of Dose Proportionality: Review of Current Approaches and Proposal for a Practical Criterion. Pharm. Stat. 8 (1), 38–49. 10.1002/PST.326 18386766

[B15] Jia W. Du F. Liu X. Jiang R. Xu F. Yang J. (2015). Renal Tubular Secretion of Tanshinol: Molecular Mechanisms, Impact on its Systemic Exposure, and Propensity for Dose-Related Nephrotoxicity and for Renal Herb-Drug Interactions. Drug Metab. Dispos 43 (5), 669–678. 10.1124/dmd.114.062000 25710938

[B16] Kang L. Si L. Rao J. Li D. Wu Y. Wu S. (2017). Polygoni Multiflori Radix Derived Anthraquinones Alter Bile Acid Disposition in sandwich-cultured Rat Hepatocytes. Toxicol. Vitro 40, 313–323. 10.1016/j.tiv.2017.01.022 28161596

[B17] Kwon B. M. Kim S. H. Baek N. I. Lee S. I. Kim E. J. Yang J. H. (2009). Farnesyl Protein Transferase Inhibitory Components of Polygonum Multiflorum. Arch. Pharm. Res. 32 (4), 495–499. 10.1007/s12272-009-1403-y 19407965

[B18] Lang W. (1993). Pharmacokinetic-metabolic Studies with 14C-Aloe Emodin after Oral Administration to Male and Female Rats. Pharmacology 47 Suppl 1 (Suppl. 1), 110–119. 10.1159/000139849 8234417

[B19] Lei X. Chen J. Ren J. Li Y. Zhai J. Mu W. (2015). Liver Damage Associated withPolygonum multiflorumThunb.: A Systematic Review of Case Reports and Case Series. Evid.-Based Complement. Altern. Med. 2015, 459749. 10.1155/2015/459749 PMC4306360 25648693

[B20] Li H. Wang X. Liu Y. Pan D. Wang Y. Yang N. (2017). Hepatoprotection and Hepatotoxicity of Heshouwu, a Chinese Medicinal Herb: Context of the Paradoxical Effect. Food Chem. Toxicol. 108 (Pt B), 407–418. 10.1016/j.fct.2016.07.035 27484243

[B21] Li D. Yang M. Zuo Z. (2020). Overview of Pharmacokinetics and Liver Toxicities of Radix Polygoni Multiflori. Toxins (Basel) 12 (11), 729. 10.3390/toxins12110729 PMC7700391 33233441

[B22] Li G. M. Chen J. R. Zhang H. Q. Cao X. Y. Sun C. Peng F. (2021). Update on Pharmacological Activities, Security, and Pharmacokinetics of Rhein. Evid. Based Complement. Alternat Med. 2021, 4582412. 10.1155/2021/4582412 34457021 PMC8387172

[B23] Li C. (2017). Multi-compound Pharmacokinetic Research on Chinese Herbal Medicines: Approach and Methodology. Zhongguo Zhong Yao Za Zhi 42 (4), 607–617. 10.19540/j.cnki.cjcmm.2017.0016 28959826

[B24] Lin L. Ni B. Lin H. Zhang M. Li X. Yin X. (2015). Traditional Usages, Botany, Phytochemistry, Pharmacology and Toxicology of Polygonum Multiflorum Thunb.: A Review. J. Ethnopharmacol 159, 158–183. 10.1016/j.jep.2014.11.009 25449462 PMC7127521

[B25] Lin C. L. Jeng J. H. Wu C. C. Hsieh S. L. Huang G. C. Leung W. (2017). Chemopreventive Potential of 2,3,5,4'-Tetrahydroxystilbene-2-O-β-D-Glucoside on the Formation of Aberrant Crypt Foci in Azoxymethane-Induced Colorectal Cancer in Rats. Biomed. Res. Int. 2017, 3634915. 10.1155/2017/3634915 29238715 PMC5697369

[B26] Liu W. Tang L. Ye L. Cai Z. Xia B. Zhang J. (2010). Species and Gender Differences Affect the Metabolism of Emodin via Glucuronidation. AAPS J. 12 (3), 424–436. 10.1208/s12248-010-9200-6 20467923 PMC2895442

[B27] Liu W. Feng Q. Li Y. Ye L. Hu M. Liu Z. (2012). Coupling of UDP-Glucuronosyltransferases and Multidrug Resistance-Associated Proteins Is Responsible for the Intestinal Disposition and Poor Bioavailability of Emodin. Toxicol. Appl. Pharmacol. 265 (3), 316–324. 10.1016/j.taap.2012.08.032 22982073

[B28] Liu M. Gong X. Quan Y. Zhou Y. Li Y. Peng C. (2018). A Cell-Based Metabonomics Approach to Investigate the Varied Influences of Chrysophanol-8-O-β-D-Glucoside with Different Concentrations on L-02 Cells. Front. Pharmacol. 9, 1530. 10.3389/fphar.2018.01530 30687094 PMC6333758

[B58] Liu W. Huang J. Zhang F. Zhang C.-C. Li R.-S. Wang Y.-L. (2021). Comprehensive Profiling and Characterization of the Absorbed Components and Metabolites in Mice Serum and Tissues Following Oral Administration of Qing-Fei-Pai-Du Decoction by UHPLC-Q-Exactive-Orbitrap HRMS. Chin. J. Nat. Medicines 19 (4), 305–320. 10.1016/s1875-5364(21)60031-6 33875170

[B61] Liu X. Yang C. Deng Y. Liu P. Yang H. Du X. (2021). Polygoni Multiflori Radix Preparat Delays Skin Aging by Inducing Mitophagy. Biomed. Res. Int. 2021, 5847153. 10.1155/2021/5847153 33511202 PMC7822667

[B29] Liu Y. Wang W. Sun M. Ma B. Pang L. Du Y. (2019). Polygonum Multiflorum-Induced Liver Injury: Clinical Characteristics, Risk Factors, Material Basis, Action Mechanism and Current Challenges. Front. Pharmacol. 10, 1–15. 10.3389/fphar.2019.01467 31920657 PMC6923272

[B30] Lu Y. Suh S. J. Li X. Liang J. L. Chi M. Hwangbo K. (2012). Citreorosein Inhibits Production of Proinflammatory Cytokines by Blocking Mitogen Activated Protein Kinases, Nuclear Factor-κB and Activator Protein-1 Activation in Mouse Bone Marrow-Derived Mast Cells. Biol. Pharm. Bull. 35 (6), 938–945. 10.1248/bpb.35.938 22687535

[B31] Lv G. P. Meng L. Z. Han D. Q. Li H. Y. Zhao J. Li S. P. (2015). Effect of Sample Preparation on Components and Liver Toxicity of Polygonum Multiflorum. J. Pharm. Biomed. Anal. 109, 105–111. 10.1016/j.jpba.2015.02.029 25766851

[B32] Ma Y. S. Weng S. W. Lin M. W. Lu C. C. Chiang J. H. Yang J. S. (2012). Antitumor Effects of Emodin on LS1034 Human colon Cancer Cells *In Vitro* and *In Vivo*: Roles of Apoptotic Cell Death and LS1034 Tumor Xenografts Model. Food Chem. Toxicol. 50 (5), 1271–1278. 10.1016/j.fct.2012.01.033 22321733

[B33] Mei-Juan L. I. Wang H. S. Wang T. B. Lai C. Leng C. L. (2018). Effect of Emodin from Polygonum Multiflori Radix Praeparata on JAK2/STAT3 Pathways in ApoE∼(-/-) Mice Atherosclerosis Model. Chin. J. Exp. Tradit. Med. Formulae 24 (18). 10.13422/j.cnki.syfjx.20181823

[B34] Park M. Y. Kwon H. J. Sung M. K. (2009). Intestinal Absorption of Aloin, Aloe-Emodin, and Aloesin; A Comparative Study Using Two *In Vitro* Absorption Models. Nutr. Res. Pract. 3 (1), 9–14. 10.4162/nrp.2009.3.1.9 20016696 PMC2788160

[B35] Pelkonen O. Pasanen M. Tolonen A. Koskinen M. Hakkola J. Abass K. (2015). Reactive Metabolites in Early Drug Development: Predictive *In Vitro* Tools. Curr. Med. Chem. 22 (4), 538–550. 10.2174/0929867321666141012175543 25312212

[B36] Qian J. Hou M. Wu X. Dai C. Sun J. Dong L. (2020). A Review on the Extraction, Purification, Detection, and Pharmacological Effects of 2,3,5,4'-Tetrahydroxystilbene-2-O-β-D-Glucoside from Polygonum Multiflorum. Biomed. Pharmacother. 124, 109923. 10.1016/j.biopha.2020.109923 31986418

[B76] Qi L. I. Zhao K. J. Zhao Y. L. (2013). High Dosage Administration of Polygonum multiflorum Alcohol Extract Caused the Multi-Organ Injury in Rats. Global J. Tradit. Chin. Med. 6 (1), 1–7. 10.3969/j.issn.1674-1749.2013.01.001

[B37] Reagan-Shaw S. Nihal M. Ahmad N. (2008). Dose Translation from Animal to Human Studies Revisited. FASEB J. 22 (3), 659–661. 10.1096/fj.07-9574LSF 17942826

[B38] Ronghua Z. Song M. Wang W. Lin P. Zhao R. (2016). Chronic Toxicity of Both Raw and Processed Polygoni Multiflori Radix on Rats. J.Chin.Phram.Sci. 25 (1), 46–56. 10.5246/jcps.2016.01.006

[B39] Ruan L. Li G. Zhao W. Meng H. Zheng Q. Wang J. (2021). Activation of Adenosine A1 Receptor in Ischemic Stroke: Neuroprotection by Tetrahydroxy Stilbene Glycoside as an Agonist. Antioxidants (Basel) 10 (7), 1–27. 10.3390/ANTIOX10071112 PMC8301086 34356346

[B40] Ruili Y. U. Men W. Zhou K. Yingli Y. U. (2019). Research Progress on Toxic Material Basis and Hepatotoxicity Mechanism of Polygonum Multiflorum. Chin. J. Pharmacovigilance 16 (8), 496–503. 10.19803/j.1672-8629.2019.08.007

[B41] Sethuraman V. S. Leonov S. Squassante L. Mitchell T. R. Hale M. D. (2007). Sample Size Calculation for the Power Model for Dose Proportionality Studies. Pharm. Stat. 6 (1), 35–41. 10.1002/PST.241 17323313

[B42] Smith B. P. Vandenhende F. R. DeSante K. A. Farid N. A. Welch P. A. Callaghan J. T. (2000). Confidence Interval Criteria for Assessment of Dose Proportionality. Pharm. Res. 17 (10), 1278–1283. 10.1023/A:1026451721686 11145235

[B43] Sun J. H. Yuan Z. F. Wang C. Y. Hui-Jun X. U. Zhang L. T. (2005). Pharmacokinetics of Stilbene Glycoside from Polygonum Multiflorum in Rats *In Vivo* . Chin. Traditional Herbal Drugs 36 (03), 405–408. 10.3321/j.issn:0253-2670.2005.03.034

[B44] Sun F. L. Zhang L. Zhang R. Y. Li L. (2011). Tetrahydroxystilbene Glucoside Protects Human Neuroblastoma SH-SY5Y Cells against MPP+-induced Cytotoxicity. Eur. J. Pharmacol. 660 (2-3), 283–290. 10.1016/j.ejphar.2011.03.046 21497157

[B45] Sun Y. N. Li W. Kim J. H. Yan X. T. Kim J. E. Yang S. Y. (2015). Chemical Constituents from the Root of Polygonum Multiflorum and Their Soluble Epoxide Hydrolase Inhibitory Activity. Arch. Pharm. Res. 38 (6), 998–1004. 10.1007/s12272-014-0520-4 25413971

[B46] Tang W. Li S. Liu Y. Huang M.-T. Ho C.-T. Ho C. T. (2017). Anti-inflammatory Effects of Trans -2,3,5,4′-tetrahydroxystilbene 2- O - β -glucopyranoside (THSG) from Polygonum Multiflorum (PM) and Hypoglycemic Effect of Cis -THSG Enriched PM Extract. J. Funct. Foods 34, 1–6. 10.1016/j.jff.2017.04.014

[B74] Trybus W. Krol T. Trybus E. Stachurska A. Krol G. (2021). The Potential Antitumor Effect of Chrysophanol in Relation to Cervical Cancer Cells. J. Cell Biochem. 122 (6), 639–652. 10.1002/jcb.29891 33417255

[B11] U.S. Department of Health and Human Services, Food and Drug Administration, Center for Drug Evaluation and Research, Center for Veterinary Medicine (2018). Bioanalytical Method Validation Guidance for Industry. Office of Medical Products and Tobacco, Center for Drug Evaluation and Research Office of Foods and Veterinary Medicine, Center for Veterinary Medicine. Available at: https://www.fda.gov/media/70858/download (February 2, 2022).

[B47] Wang Y. Yang X. W. (2008). Intestinal Transport of Free Anthraquinones in Caco-2 Cell Model. Chin. J. Nat. Medicines 6 (2), 141–145. 10.1016/S1875-5364(09)60012-110.3724/sp.j.1009.2008.00141

[B48] Wang Q. X. Wu C. Q. Yang H. L. Jing S. F. Liao M. Y. (2007). Cytotoxicity of free anthraquinone from Radix et Rhizoma Rheito HK-2 Cells. Chin. J. New Drugs 16 (03), 189–199. 10.3321/j.issn:1003-3734.2007.03.003

[B49] Wang X. Zhao L. Han T. Chen S. Wang J. (2008). Protective Effects of 2,3,5,4'-Tetrahydroxystilbene-2-O-Beta-D-Glucoside, an Active Component of Polygonum Multiflorum Thunb, on Experimental Colitis in Mice. Eur. J. Pharmacol. 578 (2-3), 339–348. 10.1016/j.ejphar.2007.09.013 17963744

[B50] Wang C. Y. Guo D. Yuan Z. F. Feng X. Zhang L. (2009). Metabolism of Stilbene Glycoside in Rats and *In Vitro* . Chin. J. Pharm. 40, 120–123. 10.3969/j.issn.1001-8255.2009.02.015

[B51] Wang P. Meng X. L. Wang J. R. Liu H. Yang Y. M. Liu R. (2011). Intestinal Absorption Kinetics of Rhubarb Mixture Free Anthraquinones in Rats. Lishizhen Med. Materia Med. Res. 22 (4), 790–792. 10.1007/s10008-010-1224-4

[B52] Wang T. Yang Y. J. Wu P. F. Wang W. Hu Z. L. Long L. H. (2011). Tetrahydroxystilbene Glucoside, a Plant-Derived Cognitive Enhancer, Promotes Hippocampal Synaptic Plasticity. Eur. J. Pharmacol. 650 (1), 206–214. 10.1016/j.ejphar.2010.10.002 20951128

[B53] Wang Y. Xu C. Wang P. Lin X. Yang Y. Li D. (2013). Pharmacokinetic Comparisons of Different Combinations of Shaoyao-Gancao-Decoction in Rats: Simultaneous Determination of Ten Active Constituents by HPLC-MS/MS. J. Chromatogr. B Analyt Technol. Biomed. Life Sci. 932, 76–87. 10.1016/j.jchromb.2013.06.021 23831700

[B54] Wang T. Wang J. Y. Zhou Z. X. Jiang Z. Z. Li Y. Y. Zhang L. (2015). Study on Hepatotoxicity of Aqueous Extracts of Polygonum Multiflorum in Rats after 28-day Oral Administration: Cholestasis-Related Mechanism. Zhongguo Zhong Yao Za Zhi 40 (11), 2163–2167. 10.4268/cjcmm20151118 26552174

[B55] Wang M. X. Wang Y. G. Huan-Hua X. U. Zhang Z. Y. Zeng-Chun M. A. Xiao C. R. (2016). Effects of Emodin in Polygonum Multiflorum on Liver Cytotoxicity and CYP450 Isoenzymes Expression in L02 Cells. Chin. Pharmacol. Bull. 32 (11), 1543–1548. 10.3969/j.issn.1001-1978.2016.11.013

[B56] Wang C. Zhou Y. Gong X. Zheng L. Li Y. (2020). *In Vitro* and *In Situ* Study on Characterization and Mechanism of the Intestinal Absorption of 2,3,5,4'-Tetrahydroxy-Stilbene-2-O-β-D-Glucoside. BMC Pharmacol. Toxicol. 21 (1), 7. 10.1186/s40360-020-0384-9 31969193 PMC6977318

[B57] Way T. D. Huang J. T. Chou C. H. Huang C. H. Yang M. H. Ho C. T. (2014). Emodin Represses TWIST1-Induced Epithelial-Mesenchymal Transitions in Head and Neck Squamous Cell Carcinoma Cells by Inhibiting the β-catenin and Akt Pathways. Eur. J. Cancer 50 (2), 366–378. 10.1016/j.ejca.2013.09.025 24157255

[B59] Wu L. Han W. Chen Y. Zhang T. Liu J. Zhong S. (2018). Gender Differences in the Hepatotoxicity and Toxicokinetics of Emodin: The Potential Mechanisms Mediated by UGT2B7 and MRP2. Mol. Pharm. 15 (9), 3931–3945. 10.1021/acs.molpharmaceut.8b00387 30011215

[B60] Xiang K. Liu G. Zhou Y. J. Hao H. Z. Yin Z. He A. D. (2014). 2,3,5,4'-tetrahydroxystilbene-2-O-β-D-glucoside (THSG) Attenuates Human Platelet Aggregation, Secretion and Spreading *In Vitro* . Thromb. Res. 133 (2), 211–217. 10.1016/j.thromres.2013.11.006 24332167

[B62] Yang X.-P. Liu T. Y. Qin X. Y. Yu L. C. (2014). Potential protection of 2,3,5,4'-Tetrahydroxystilbene-2-O-β-D-Glucoside against Staurosporine-Induced Toxicity on Cultured Rat hippocampus Neurons. Neurosci. Lett. 576, 79–83. 10.1016/j.neulet.2014.05.045 24887581

[B63] Xing Y. Wang L. Wang C. Zhang Y. Zhang Y. Hu L. (2019). Pharmacokinetic Studies Unveiled the Drug-Drug Interaction between Trans-2,3,5,4'-tetrahydroxystilbene-2-O-β-d-glucopyranoside and Emodin that May Contribute to the Idiosyncratic Hepatotoxicity of Polygoni Multiflori Radix. J. Pharm. Biomed. Anal. 164, 672–680. 10.1016/j.jpba.2018.11.034 30472586

[B64] Yang X. Kang M. C. Li Y. Kim E. A. Kang S. M. Jeon Y. J. (2014). Anti-inflammatory Activity of Questinol Isolated from marine-derived Fungus Eurotium amstelodami in Lipopolysaccharide-Stimulated RAW 264.7 Macrophages. J. Microbiol. Biotechnol. 24 (10), 1346–1353. 10.4014/jmb.1405.05035 24986678

[B73] Xie W. Zhao Y. Du L. (2012). Emerging Approaches of Traditional Chinese Medicine Formulas for the Treatment of Hyperlipidemia. J. Ethnopharmacol. 140 (2), 345–367. 10.1016/j.jep.2012.01.027 22306102

[B65] Xu X. L. Huang Y. J. Chen X. F. Lin D. Y. Zhang W. (2012). 2,3,4',5-tetrahydroxystilbene-2-O-β-D-glucoside Inhibits Proliferation of Vascular Smooth Muscle Cells: Involvement of NO/cGMP/PKG Pathway. Phytother Res. 26 (7), 1068–1074. 10.1002/ptr.3691 22213189

[B66] Yang M. Liu T. Feng W. H. Hui L. Q. Li R. R. Liu X. Q. (2016). Exploration Research on Hepatotoxic Constituents from Polygonum Multiflorum Root. Zhongguo Zhong Yao Za Zhi 41 (7), 1289–1296. 10.4268/cjcmm20160721 28879745

[B67] Yao W. Fan W. Huang C. Zhong H. Chen X. Zhang W. (2013). Proteomic Analysis for Anti-atherosclerotic Effect of Tetrahydroxystilbene Glucosi- Interaction between Trans-2,3,5,4'-tetrahydroxystilbene-2-O-β-d-glucopyranoside and Emodin that May Contribute to the Idiosyncratic Hepatotoxicity of Polygoni Multiflori Radix. J. Pharm. Biomed. Anal. 164, 672–680. 10.1016/j.jpba.2018.11.034 30472586

[B68] Yao W. Gu C. Shao H. Meng G. Wang H. Jing X. (2015). Tetrahydroxystilbene Glucoside Improves TNF-α-Induced Endothelial Dysfunction: Involvement of TGFβ/Smad Pathway and Inhibition of Vimentin Expression. Am. J. Chin. Med. 43 (1), 183–198. 10.1142/s0192415x15500123 25571766

[B69] Yuan T. Liu X. (2016). Fleece-Flower Root Extract Diphenylethylene Glycosides in Diabetic Renal Tubular Injury Protection Experimental Study. Chin. J. Integr. Tradit. West. Nephrol. 17 (2), 114–118. CNKI:SUN:JXSB.0.2016-02-009.

[B70] Zhang C. Zhang R. C. Sun Z. X. (2013). Study on the Hepatoxicity of Polygoni Multiflori Radix and Polygoni Multiflori Rodix Praeparata in Rats. Zhong Yao Cai 36 (9), 1416–1419. 10.13863/j.issn1001-4454.2013.09.017 24620682

[B71] Zhang M. Lin L. Lin H. Qu C. Yan L. Ni J. (2018). Interpretation the Hepatotoxicity Based on Pharmacokinetics Investigated through Oral Administrated Different Extraction Parts of Polygonum Multiflorum on Rats. Front. Pharmacol. 9 (505), 505–513. 10.3389/fphar.2018.00505 29887801 PMC5980962

[B72] Zhu Y. Liu S. H. Wang J. B. Song H. B. Li Y. G. He T. T. (2015). Clinical Analysis of Drug-Induced Liver Injury Caused by Polygonum Multiflorum and its Preparations. Zhongguo Zhong Xi Yi Jie He Za Zhi 35 (12), 1442–1447. 10.7661/CJIM.2015.12.1442 26882605

