# Supplementary material for: Pharmacokinetics, Tissue Distribution, and Excretion Characteristics of a Radix Polygoni Multiflori Extract in Rats
Source: Front Pharmacol. 2022 Feb 21;13:827668. doi: 10.3389/fphar.2022.827668 (PMC8899820; doi:10.3389/fphar.2022.827668)
Supplement: Supplementary file 1 [file DataSheet1.docx]

Supplementary Material

**Supplemental LC-MS/MS instrument and analytical conditions**

Chromatographic separation was achieved on a waters HSS-T3 C18 column (100 × 2.1mm, 1.8 mm, kept at 40 °C) using a mobile phase containing 0.025% formic acid that consisted of solvent A (water) and solvent B (acetonitrile). The mobile phase was delivered at 0.3 mL/min, and the gradient program shown in Table S1 was used (Cheng et al., 2020).

The electrospray ionization (ESI) source of the mass spectrometer was operated in the negative ion mode. The ion source parameters were optimized as follows: turbo spray temperature, 500 °C; nebulizer gas (gas 1), 45 psi; heater gas (gas 2), 50 psi; and curtain gas 45 psi. The dwell time was 50 ms for all analytes. The entrance potential (EP) and collision exit potential (CXP) were set at –10 V and –16 V, respectively. The declustering potential (DP), collision energy (CE) and other detailed mass spectrometry conditions by Cheng et al., 2020.

**Table S1** Liquid phase elution conditions of RPM compounds.

| **Time**  **(min)** | **A**  **(%)** | **B**  **(%)** | **Curve** |
| --- | --- | --- | --- |
| initial | 95 | 5 | initial |
| 1 | 75 | 25 | 6 |
| 3.5 | 65 | 35 | 6 |
| 5 | 60 | 40 | 7 |
| 6 | 40 | 60 | 6 |
| 6.1 | 5 | 95 | 6 |
| 8 | 0 | 100 | 6 |
| 9 | 95 | 5 | 1 |

**Supplemental Rat Tissue Methodological verification**

**Specificity and selectivity** Six sources of blank rat liver were selected and processed according to the “*2.5 Standard and Sample Preparation*” and then analyzed by UPLC-MS/MS to investigate the selectivity of the method.

**Accuracy and precision** Prepare rat liver samples with 4 levels of LLOQ, quality control concentration low, medium, and high, respectively, and each concentration is 6 samples in parallel to investigate the accuracy and precision of the analysis method of each component of Polygonum multiflorum in rat plasma samples.

***Results***

**Specificity and selectivity** Under the conditions described above, all rat liver samples showed interfering peak in their peak region, indicating the substitution and selectivity of detailed steps.

**Figure S2 (1)** Representative chromatogram of the 13 constituents and IS in blank rats liver, LLOQ and real samples.

**Figure S2 (2)** Representative chromatogram of the 13 constituents and IS in blank rats liver, LLOQ and real samples.

**Precision and Accuracy** The intra-day accuracy and precision (% RSD) data for the 13 components were presented in *Table S2*. The average intraday accuracy is within 15%, and the intraday precision RSD values are all less than 15%.

**Table S2** Accuracy and precision of 13 constituents in rat liver. (n=6)

| **Compound** | **Nominal concentration (ng/mL)** | **Intra-day** | | |
| --- | --- | --- | --- | --- |
|  |  | **Measured concentration（ng/mL）** | **Accuracy (%)** | **Precision (%)** |
| Chrysophanol | 1 | 1.02 ± 0.074 | 102 | 7.27 |
|  | 2 | 2.10 ± 0.128 | 105 | 6.10 |
|  | 27 | 27.3 ± 2.52 | 101 | 9.25 |
|  | 583 | 576 ± 53.0 | 98.8 | 9.20 |
| Emodin | 0.1 | 0.105 ± 0.007 | 105 | 6.51 |
|  | 0.2 | 0.214 ± 0.012 | 107 | 5.64 |
|  | 2.7 | 2.82 ± 0.161 | 104 | 5.72 |
|  | 58.3 | 59.3 ± 5.57 | 102 | 9.39 |
| Aloe-emodin | 0.1 | 0.98 ± 0.009 | 98.1 | 8.72 |
|  | 0.2 | 0.190 ± 0.007 | 95.2 | 3.56 |
|  | 2.7 | 2.61 ± 0.214 | 96.7 | 8.19 |
|  | 58.3 | 57.4 ± 4.27 | 98.4 | 7.43 |
| Rhein | 0.5 | 0.507 ± 0.053 | 101 | 10.4 |
|  | 1 | 1.09 ± 0.025 | 109 | 2.29 |
|  | 13.5 | 13.8 ± 1.43 | 102 | 10.4 |
|  | 292 | 275 ± 30.8 | 94.0 | 11.2 |
| Physcion | 0.5 | 0.504 ± 0.047 | 101 | 9.23 |
|  | 1 | 0.934 ± 0.011 | 93.4 | 1.14 |
|  | 13.5 | 14.2 ± 0.748 | 105 | 5.28 |
|  | 292 | 277 ± 13.2 | 94.9 | 4.77 |
| Questin | 0.1 | 0.104 ± 0.005 | 104 | 5.07 |
|  | 0.2 | 0.214 ± 0.011 | 107 | 5.01 |
|  | 2.7 | 2.68 ± 0.151 | 99.3 | 5.65 |
|  | 58.3 | 54.4 ± 2.50 | 93.3 | 4.59 |
| Citreorosein | 0.1 | 0.103 ± 0.010 | 103 | 9.81 |
|  | 0.2 | 0.196 ± 0.021 | 98.1 | 10.7 |
|  | 2.7 | 2.76 ± 0.167 | 102 | 6.04 |
|  | 58.3 | 61.1 ± 4.48 | 105 | 7.32 |
| Questinol | 0.1 | 0.109 ± 0.006 | 109 | 5.37 |
|  | 0.2 | 0.197 ± 0.015 | 98.5 | 7.62 |
|  | 2.7 | 2.89 ± 0.169 | 107 | 5.85 |
|  | 58.3 | 54.7 ± 3.22 | 93.8 | 5.89 |
| TSG | 0.3 | 0.311 ± 0.028 | 104 | 8.87 |
|  | 0.6 | 0.574 ± 0.030 | 95.7 | 5.22 |
|  | 8.1 | 7.69 ± 0.724 | 94.9 | 9.41 |
|  | 175 | 175 ± 11.1 | 100 | 6.32 |
| TG | 0.1 | 0.100 ± 0.008 | 100 | 8.40 |
|  | 0.2 | 0.202 ± 0.014 | 101 | 7.07 |
|  | 2.7 | 2.59 ± 0.058 | 95.8 | 2.15 |
|  | 58.3 | 58.8 ± 3.45 | 101 | 5.86 |
| CG | 0.1 | 0.105 ± 0.005 | 105 | 5.00 |
|  | 0.2 | 0.208 ± 0.011 | 104 | 5.26 |
|  | 2.7 | 2.85 ± 0.103 | 105 | 3.83 |
|  | 58.3 | 59.9 ± 3.80 | 103 | 6.51 |
| EG | 0.1 | 0.101 ± 0.003 | 101 | 3.17 |
|  | 0.2 | 0.204 ± 0.012 | 102 | 6.31 |
|  | 2.7 | 2.63 ± 0.123 | 97.4 | 4.56 |
|  | 58.3 | 60.3 ± 2.30 | 103 | 3.95 |
| PG | 0.1 | 0.096 ± 0.005 | 96.0 | 5.10 |
|  | 0.2 | 0.206 ± 0.009 | 103 | 4.51 |
|  | 2.7 | 2.73 ± 0.276 | 101 | 10.2 |
|  | 58.3 | 57.8 ± 4.23 | 99.1 | 7.26 |

**Supplemental Rat Bile, Urine and Feces Methodological verification**

**Specificity and selectivity** Six sources of blank rat urine, feces and bile samples were selected and processed according to the “*2.5 Standard and Sample Preparation*” and then analyzed by UPLC-MS/MS to investigate the selectivity of the method.

**Accuracy and precision** Prepare rat urine, feces and bile samples with 4 levels of LLOQ, quality control concentration low, medium, and high, respectively, and each concentration is 6 samples in parallel to investigate the accuracy and precision of the analysis method of each component of Polygonum multiflorum in rat plasma samples.

***Results***

**Specificity and selectivity** Under the conditions described above, all rat urine, feces and bile samples showed that there was no interfering peak in their peak region, indicating the substitution and selectivity of detailed steps.

**Figure S2 (1)** Representative chromatograms of the 13 constituents and IS, including of blank rats urine sample, LLOQ and real samples.

**Figure S2 (2)** Representative chromatograms of the 13 constituents and IS, including of blank rats urine sample, LLOQ and real samples.

**Figure S3 (1)** Representative chromatograms of the 13 constituents and IS, including of blank rats feces sample, LLOQ and real samples.

**Figure S3 (2)** Representative chromatograms of the 13 constituents and IS, including of blank rats feces sample, LLOQ and real samples.

**Figure S4 (1)** Representative chromatograms of the 13 constituents and IS, including of blank rats bile sample, LLOQ and real samples.

**Figure S4 (2)** Representative chromatograms of the 13 constituents and IS, including of blank rats bile sample, LLOQ and real samples.

**Precision and Accuracy** The intra-day and inter-day accuracy and precision of the components of Polygonum multiflorum extract in urine, feces and bile were all within the standard range, and the intra-day accuracy and precision of each compound in urine were 93.3–108% and 2.83–12.8%, the inter-day accuracy and precision were 89.9–110% and 1.48–10.2%; the intra-day accuracy and precision in feces were 91.1–109% and 1.96–10.7%, respectively, the inter-day accuracy and precision were 91.3–109% and 2.13–10.7%, respectively; the intra-day accuracy and precision in bile were 89.8–107% and 2.85–11.2%, and the inter-day accuracy and precision are respectively 93.3–108% and 2.72–11.0%.

**Table S3** Accuracy and precision of 13 constituents in rat urine.

| **Compound** | **Nominal concentration (ng/mL)** | **Intra-day (n=6)** | | | **Inter-day (n=18)** | | |
| --- | --- | --- | --- | --- | --- | --- | --- |
|  |  | **Measured concentration**  **（ng/mL）** | **Accuracy**  **(%)** | **Precision**  **(%)** | **Measured concentration**  **（ng/mL）** | **Accuracy**  **(%)** | **Precision**  **(%)** |
| Chrysophanol | 1 | 0.950 ± 0.042 | 95.2 | 4.42 | 1.10 ± 0.048 | 110 | 4.32 |
|  | 2 | 2.00 ± 0.153 | 100 | 7.64 | 2.02 ± 0.190 | 101 | 9.41 |
|  | 27 | 28.2 ± 1.93 | 104 | 6.86 | 28.1 ± 1.71 | 104 | 6.07 |
|  | 583 | 600 ± 48.7 | 103 | 8.11 | 576 ± 28.5 | 98.7 | 4.95 |
| Emodin | 0.1 | 0.097 ± 0.007 | 97.0 | 6.71 | 0.098 ± 0.005 | 97.9 | 5.01 |
|  | 0.2 | 0.197 ± 0.018 | 98.7 | 9.26 | 0.206 ± 0.011 | 103 | 5.32 |
|  | 2.7 | 2.77 ± 0.190 | 103 | 6.85 | 2.66 ± 0.171 | 98.3 | 6.45 |
|  | 58.3 | 55.8 ± 3.68 | 95.8 | 6.60 | 56.8 ± 4.06 | 97.5 | 7.15 |
| Aloe-emodin | 0.1 | 0.098 ± 0.008 | 97.9 | 7.67 | 0.093 ± 0.004 | 93.2 | 4.02 |
|  | 0.2 | 0.195 ± 0.016 | 97.6 | 8.43 | 0.188 ± 0.006 | 94.1 | 2.93 |
|  | 2.7 | 2.56 ± 0.139 | 94.8 | 5.42 | 2.51 ± 0.146 | 93.1 | 5.80 |
|  | 58.3 | 60.8 ± 3.58 | 104 | 5.89 | 60.0 ± 4.61 | 103 | 7.69 |
| Rhein | 0.5 | 0.507 ± 0.046 | 101 | 9.16 | 0.482 ± 0.036 | 96.3 | 7.38 |
|  | 1 | 1.06 ± 0.041 | 106 | 3.89 | 0.966 ± 0.098 | 96.6 | 10.2 |
|  | 13.5 | 14.3 ± 0.813 | 106 | 5.70 | 14.2 ± 1.02 | 106 | 7.16 |
|  | 292 | 287 ± 32.8 | 98.2 | 11.4 | 287 ± 28.6 | 98.2 | 9.97 |
| Physcion | 0.5 | 0.499 ± 0.040 | 99.8 | 7.91 | 0.520 ± 0.028 | 104 | 5.51 |
|  | 1 | 0.951 ± 0.086 | 95.1 | 9.05 | 0.899 ± 0.042 | 89.9 | 4.64 |
|  | 13.5 | 13.1 ± 1.15 | 97.0 | 8.80 | 14.0 ± 1.17 | 104 | 8.40 |
|  | 292 | 279 ± 35.8 | 95.5 | 12.8 | 307 ± 28.7 | 105 | 9.35 |
| Questin | 0.1 | 0.097 ± 0.004 | 97.2 | 4.17 | 0.096 ± 0.005 | 96.0 | 5.46 |
|  | 0.2 | 0.203 ± 0.013 | 102 | 6.61 | 0.206 ± 0.019 | 103 | 9.15 |
|  | 2.7 | 2.58 ± 0.146 | 95.7 | 5.64 | 2.86 ± 0.135 | 106 | 4.73 |
|  | 58.3 | 58.7 ± 3.38 | 101 | 5.79 | 56.7 ± 4.54 | 97.3 | 7.99 |
| Citreorosein | 0.1 | 0.097 ± 0.006 | 97.1 | 6.39 | 0.105 ± 0.005 | 105 | 4.80 |
|  | 0.2 | 0.203 ± 0.010 | 102 | 4.98 | 0.195 ± 0.006 | 97.5 | 3.14 |
|  | 2.7 | 2.63 ± 0.202 | 97.6 | 7.68 | 2.76 ± 0.148 | 102 | 5.38 |
|  | 58.3 | 58.2 ± 3.48 | 99.9 | 5.98 | 55.9 ± 2.34 | 96.0 | 4.18 |
| Questinol | 0.1 | 0.104 ± 0.005 | 104 | 4.43 | 0.107 ± 0.008 | 107 | 7.25 |
|  | 0.2 | 0.204 ± 0.010 | 102 | 4.69 | 0.186 ± 0.005 | 92.9 | 2.71 |
|  | 2.7 | 2.80 ± 0.219 | 104 | 7.83 | 2.83 ± 0.214 | 105 | 7.59 |
|  | 58.3 | 55.6 ± 2.34 | 95.4 | 4.20 | 59.6 ± 2.96 | 102 | 4.97 |
| TSG | 0.3 | 0.296 ± 0.0163 | 98.6 | 5.52 | 0.306 ± 0.023 | 102 | 7.48 |
|  | 0.6 | 0.561 ± 0.031 | 93.5 | 5.51 | 0.595 ± 0.046 | 99.2 | 7.65 |
|  | 8.1 | 7.55 ± 0.430 | 93.3 | 5.70 | 8.39 ± 0.505 | 104 | 6.01 |
|  | 175 | 168 ± 8.19 | 96.2 | 4.87 | 169 ± 12.1 | 96.4 | 7.16 |
| TG | 0.1 | 0.099 ± 0.007 | 99.0 | 6.94 | 0.096 ± 0.005 | 96.2 | 4.81 |
|  | 0.2 | 0.197 ± 0.006 | 98.3 | 2.84 | 0.210 ± 0.010 | 105 | 4.57 |
|  | 2.7 | 2.64 ± 0.112 | 97.6 | 4.17 | 2.79 ± 0.152 | 103 | 5.46 |
|  | 58.3 | 57.1 ± 1.62 | 97.9 | 2.83 | 57.9 ± 1.80 | 99.3 | 3.11 |
| CG | 0.1 | 0.097 ± 0.006 | 97.4 | 5.75 | 0.106 ± 0.003 | 106 | 2.77 |
|  | 0.2 | 0.195 ± 0.012 | 97.6 | 5.98 | 0.211 ± 0.006 | 105 | 2.80 |
|  | 2.7 | 2.74 ± 0.222 | 102 | 8.09 | 2.49 ± 0.086 | 92.4 | 3.46 |
|  | 58.3 | 60.9 ± 3.74 | 104 | 6.14 | 58.6 ± 3.22 | 101 | 5.50 |
| Questinol | 0.1 | 0.104 ± 0.005 | 104 | 4.43 | 0.107 ± 0.008 | 107 | 7.25 |
|  | 0.2 | 0.204 ± 0.010 | 102 | 4.69 | 0.186 ± 0.005 | 92.9 | 2.71 |
|  | 2.7 | 2.80 ± 0.219 | 104 | 7.83 | 2.83 ± 0.214 | 105 | 7.59 |
|  | 58.3 | 55.6 ± 2.34 | 95.4 | 4.20 | 59.6 ± 2.96 | 102 | 4.97 |
| TSG | 0.3 | 0.296 ± 0.0163 | 98.6 | 5.52 | 0.306 ± 0.023 | 102 | 7.48 |
|  | 0.6 | 0.561 ± 0.031 | 93.5 | 5.51 | 0.595 ± 0.046 | 99.2 | 7.65 |
|  | 8.1 | 7.55 ± 0.430 | 93.3 | 5.70 | 8.39 ± 0.505 | 104 | 6.01 |
|  | 175 | 168 ± 8.19 | 96.2 | 4.87 | 169 ± 12.1 | 96.4 | 7.16 |
| TG | 0.1 | 0.099 ± 0.007 | 99.0 | 6.94 | 0.096 ± 0.005 | 96.2 | 4.81 |
|  | 0.2 | 0.197 ± 0.006 | 98.3 | 2.84 | 0.210 ± 0.010 | 105 | 4.57 |
|  | 2.7 | 2.64 ± 0.112 | 97.6 | 4.17 | 2.79 ± 0.152 | 103 | 5.46 |
|  | 58.3 | 57.1 ± 1.62 | 97.9 | 2.83 | 57.9 ± 1.80 | 99.3 | 3.11 |
| CG | 0.1 | 0.097 ± 0.006 | 97.4 | 5.75 | 0.106 ± 0.003 | 106 | 2.77 |
|  | 0.2 | 0.195 ± 0.012 | 97.6 | 5.98 | 0.211 ± 0.006 | 105 | 2.80 |
|  | 2.7 | 2.74 ± 0.222 | 102 | 8.09 | 2.49 ± 0.086 | 92.4 | 3.46 |
|  | 58.3 | 60.9 ± 3.74 | 104 | 6.14 | 58.6 ± 3.22 | 101 | 5.50 |
| EG | 0.1 | 0.094 ± 0.005 | 93.6 | 4.89 | 0.099 ± 0.005 | 98.7 | 5.06 |
|  | 0.2 | 0.215 ± 0.010 | 108 | 4.59 | 0.199 ± 0.018 | 99.3 | 9.16 |
|  | 2.7 | 2.79 ± 0.167 | 103 | 5.99 | 2.80 ± 0.106 | 104 | 3.78 |
|  | 58.3 | 57.7 ± 4.57 | 98.9 | 7.92 | 53.8 ± 2.42 | 92.3 | 4.50 |
| PG | 0.1 | 0.099 ± 0.005 | 98.8 | 4.95 | 0.103 ± 0.005 | 103 | 4.56 |
|  | 0.2 | 0.208 ± 0.010 | 104 | 4.67 | 0.206 ± 0.003 | 103 | 1.48 |
|  | 2.7 | 2.74 ± 0.103 | 101 | 3.81 | 2.52 ± 0.104 | 93.3 | 3.83 |
|  | 58.3 | 57.5 ± 2.37 | 98.7 | 4.07 | 58.9 ± 2.67 | 101 | 4.57 |

**Table S4** Accuracy and precision of 13 constituents in rat faeces.

| **Compound** | **Nominal concentration (ng/mL)** | **Intra-day (n=6)** | | | **Inter-day (n=18)** | | |
| --- | --- | --- | --- | --- | --- | --- | --- |
|  |  | **Measured concentration**  **（ng/mL）** | **Accuracy**  **(%)** | **Precision**  **(%)** | **Measured concentration**  **（ng/mL）** | **Accuracy**  **(%)** | **Precision**  **(%)** |
| Chrysophanol | 1 | 1.07 ± 0.531 | 107 | 4.95 | 0.944 ± 0.026 | 94.4 | 2.80 |
|  | 2 | 2.12 ± 0.121 | 106 | 5.72 | 1.97 ± 0.089 | 98.7 | 4.53 |
|  | 27 | 28.3 ± 2.28 | 105 | 8.05 | 28.1 ± 1.57 | 104 | 5.58 |
|  | 583 | 595 ± 26.0 | 102 | 4.37 | 606 ± 33.5 | 104 | 5.53 |
| Emodin | 0.1 | 0.104 ± 0.004 | 104 | 3.99 | 0.096 ± 0.003 | 96.2 | 2.87 |
|  | 0.2 | 0.203 ± 0.010 | 102 | 5.07 | 0.187 ± 0.011 | 93.3 | 5.89 |
|  | 2.7 | 2.63 ± 0.127 | 97.4 | 4.82 | 2.81 ± 0.116 | 104 | 4.15 |
|  | 58.3 | 61.0 ± 3.35 | 105 | 5.49 | 63.2 ± 1.59 | 108 | 2.52 |
| Aloe-emodin | 0.1 | 0.093 ± 0.003 | 93.3 | 3.59 | 0.102 ± 0.006 | 102 | 5.85 |
|  | 0.2 | 0.198 ± 0.009 | 98.9 | 4.66 | 0.206 ± 0.11 | 103 | 5.15 |
|  | 2.7 | 2.73 ± 0.190 | 101 | 6.95 | 2.54 ± 0.132 | 94.0 | 5.18 |
|  | 58.3 | 58.4 ± 2.59 | 100 | 4.44 | 55.3 ± 2.75 | 94.9 | 4.96 |
| Rhein | 0.5 | 0.462 ± 0.010 | 92.4 | 2.26 | 0.485 ± 0.035 | 97.1 | 7.19 |
|  | 1 | 0.917 ± 0.041 | 91.7 | 4.49 | 0.923 ± 0.078 | 92.3 | 8.50 |
|  | 13.5 | 14.6 ± 0.508 | 108 | 3.49 | 13.5 ± 1.04 | 99.7 | 7.71 |
|  | 292 | 266 ± 20.7 | 91.1 | 7.79 | 273 ± 27.1 | 93.4 | 9.93 |
| Physcion | 0.5 | 0.513 ± 0.031 | 103 | 5.95 | 0.519 ± 0.030 | 104 | 5.69 |
|  | 1 | 0.923 ± 0.078 | 92.3 | 8.50 | 0.980 ± 0.105 | 98.0 | 10.7 |
|  | 13.5 | 13.5 ± 1.04 | 99.7 | 7.71 | 13.0 ± 0.687 | 96.3 | 5.28 |
|  | 292 | 273 ± 27.1 | 93.4 | 9.93 | 304 ± 21.8 | 104 | 7.16 |
| Questin | 0.1 | 0.097 ± 0.006 | 97.3 | 5.89 | 0.099 ± 0.008 | 98.8 | 7.61 |
|  | 0.2 | 0.206 ± 0.015 | 103 | 7.44 | 0.196 ± 0.018 | 97.9 | 8.95 |
|  | 2.7 | 2.73 ± 0.154 | 101 | 5.64 | 2.59 ± 0.148 | 95.8 | 5.73 |
|  | 58.3 | 58.9 ± 3.22 | 101 | 5.47 | 56.9 ± 2.48 | 97.6 | 4.36 |
| Citreorosein | 0.1 | 0.101 ± 0.007 | 101 | 6.88 | 0.095 ± 0.005 | 95.1 | 5.68 |
|  | 0.2 | 0.194 ± 0.014 | 96.8 | 7.10 | 0.196 ± 0.013 | 97.8 | 6.50 |
|  | 2.7 | 2.55 ± 0.165 | 94.4 | 6.47 | 2.69 ± 0.159 | 99.6 | 5.92 |
|  | 58.3 | 55.0 ± 3.64 | 94.4 | 6.62 | 53.8 ± 2.42 | 92.3 | 4.50 |
| Questinol | 0.1 | 0.109 ± 0.004 | 109 | 6.69 | 0.102 ± 0.009 | 102 | 8.66 |
|  | 0.2 | 0.193 ± 0.129 | 96.6 | 6.66 | 0.198 ± 0.009 | 98.8 | 4.63 |
|  | 2.7 | 2.74 ± 0.292 | 101 | 10.7 | 2.75 ± 0.156 | 102 | 5.68 |
|  | 58.3 | 57.8 ± 3.39 | 99.1 | 5.86 | 58.7 ± 4.74 | 101 | 8.07 |
| TSG | 0.3 | 0.289 ± 0.027 | 96.4 | 9.42 | 0.274 ± 0.007 | 91.3 | 2.47 |
|  | 0.6 | 0.582 ± 0.057 | 97.0 | 9.84 | 0.619 ± 0.040 | 103 | 6.44 |
|  | 8.1 | 7.87 ± 0.566 | 97.1 | 7.19 | 8.25 ± 0.743 | 102 | 9.01 |
|  | 175 | 174 ± 15.1 | 99.6 | 8.66 | 191 ± 4.40 | 109 | 2.31 |
| TG | 0.1 | 0.095 ± 0.006 | 94.8 | 6.78 | 0.098 ± 0.002 | 98.5 | 2.13 |
|  | 0.2 | 0.204 ± 0.007 | 102 | 3.61 | 0.193 ± 0.006 | 96.6 | 2.93 |
|  | 2.7 | 2.76 ± 0.220 | 102 | 7.97 | 2.71 ± 0.096 | 100 | 3.55 |
|  | 58.3 | 56.0 ± 2.93 | 96.0 | 5.23 | 59.7 ± 2.98 | 102 | 5.00 |
| CG | 0.1 | 0.099 ± 0.006 | 98.9 | 6.14 | 0.099 ± 0.008 | 98.7 | 7.98 |
|  | 0.2 | 0.198 ± 0.012 | 99.0 | 6.16 | 2.08 ± 0.009 | 104 | 4.53 |
|  | 2.7 | 2.73 ± 0.176 | 101 | 6.46 | 2.55 ± 0.068 | 94.6 | 2.67 |
|  | 58.3 | 59.7 ± 2.17 | 102 | 3.64 | 55.3 ± 2.37 | 94.8 | 4.29 |
| EG | 0.1 | 0.097 ± 0.009 | 97.2 | 8.96 | 0.101 ± 0.004 | 101 | 4.36 |
|  | 0.2 | 0.207 ± 0.0207 | 104 | 9.98 | 0.208 ± 0.011 | 104 | 5.25 |
|  | 2.7 | 2.63 ± 0.146 | 97.3 | 5.56 | 2.76 ± 0.059 | 102 | 2.13 |
|  | 58.3 | 55.8 ± 3.92 | 95.7 | 7.02 | 58.0 ± 1.57 | 99.5 | 2.71 |
| PG | 0.1 | 0.097 ± 0.006 | 96.8 | 5.80 | 0.107 ± 0.003 | 107 | 2.86 |
|  | 0.2 | 0.200 ± 0.011 | 100 | 5.63 | 0.198 ± 0.012 | 98.8 | 6.12 |
|  | 2.7 | 2.58 ± 0.180 | 95.5 | 6.68 | 2.73 ± 0.156 | 101 | 5.71 |
|  | 58.3 | 53.8 ± 1.14 | 92.2 | 1.96 | 55.3 ± 1.63 | 94.9 | 2.94 |

**Table S5** Accuracy and precision of 13 constituents in rat bile.

| **Compound** | **Nominal concentration (ng/mL)** | **Intra-day (n=6)** | | | **Inter-day (n=18)** | | |
| --- | --- | --- | --- | --- | --- | --- | --- |
|  |  | **Measured concentration**  **（ng/mL）** | **Accuracy**  **(%)** | **Precision**  **(%)** | **Measured concentration**  **（ng/mL）** | **Accuracy**  **(%)** | **Precision**  **(%)** |
| Chrysophanol | 1 | 0.979 ± 0.090 | 97.9 | 9.18 | 1.02 ± 0.039 | 102 | 3.86 |
|  | 2 | 1.94 ± 0.208 | 96.9 | 10.7 | 1.87 ± 0.132 | 93.3 | 7.08 |
|  | 27 | 26.6 ± 2.58 | 98.5 | 9.68 | 25.8 ± 1.74 | 95.6 | 6.75 |
|  | 583 | 621 ± 34.5 | 107 | 5.55 | 602 ± 18.5 | 103 | 3.07 |
| Emodin | 0.1 | 0.100 ± 0.010 | 99.7 | 10.4 | 0.101 ± 0.006 | 101 | 5.82 |
|  | 0.2 | 0.198 ± 0.013 | 98.8 | 6.54 | 0.209 ± 0.009 | 105 | 4.22 |
|  | 2.7 | 2.68 ± 0.197 | 99.1 | 7.35 | 2.65 ± 0.095 | 98.2 | 3.58 |
|  | 58.3 | 57.4 ± 5.09 | 98.5 | 8.86 | 59.3 ± 4.18 | 102 | 7.05 |
| Aloe-emodin | 0.1 | 0.091 ± 0.003 | 91.4 | 2.86 | 0.102 ± 0.010 | 102 | 9.97 |
|  | 0.2 | 0.200 ± 0.010 | 99.9 | 5.04 | 0.201 ± 0.014 | 101 | 7.06 |
|  | 2.7 | 2.88 ± 0.168 | 107 | 5.83 | 2.86 ± 0.155 | 106 | 5.40 |
|  | 58.3 | 60.8 ± 3.44 | 104 | 5.66 | 59.4 ± 3.77 | 102 | 6.34 |
| Rhein | 0.5 | 0.491 ± 0.027 | 98.2 | 5.42 | 0.503 ± 0.028 | 101 | 5.63 |
|  | 1 | 0.946 ± 0.070 | 94.6 | 7.42 | 0.978 ± 0.065 | 97.8 | 6.64 |
|  | 13.5 | 12.8 ± 0.963 | 94.6 | 7.54 | 12.7 ± 0.426 | 93.8 | 3.36 |
|  | 292 | 273 ± 23.0 | 93.3 | 8.44 | 276 ± 18.6 | 94.4 | 6.73 |
| Physcion | 0.5 | 0.492 ± 0.034 | 98.4 | 6.84 | 0.528 ± 0.014 | 106 | 2.72 |
|  | 1 | 0.899 ± 0.042 | 89.9 | 4.64 | 1.01 ± 0.082 | 101 | 8.07 |
|  | 13.5 | 14.0 ± 0.794 | 103 | 5.69 | 13.4 ± 1.27 | 99.0 | 9.50 |
|  | 292 | 262 ± 10.7 | 89.8 | 4.07 | 302 ± 24.0 | 104 | 7.94 |
| Questin | 0.1 | 0.096 ± 0.004 | 95.5 | 4.01 | 0.103 ± 0.003 | 103 | 3.19 |
|  | 0.2 | 0.204 ± 0.011 | 102 | 5.49 | 0.190 ± 0.011 | 95.2 | 5.90 |
|  | 2.7 | 2.57 ± 0.161 | 95.2 | 6.28 | 2.73 ± 0.200 | 101 | 7.33 |
|  | 58.3 | 59.2 ± 2.92 | 102 | 4.94 | 56.5 ± 4.68 | 96.8 | 8.30 |
| Citreorosein | 0.1 | 0.102 ± 0.005 | 102 | 4.94 | 0.098 ± 0.007 | 97.6 | 7.22 |
|  | 0.2 | 0.190 ± 0.013 | 95.0 | 6.83 | 0.207 ± 0.014 | 103 | 6.91 |
|  | 2.7 | 2.75 ± 0.227 | 102 | 8.26 | 2.86 ± 0.177 | 106 | 6.20 |
|  | 58.3 | 56.5 ± 5.34 | 96.8 | 9.46 | 60.3 ± 2.79 | 103 | 4.63 |
| Questinol | 0.1 | 0.104 ± 0.007 | 104 | 7.08 | 0.107 ± 0.005 | 107 | 4.96 |
|  | 0.2 | 0.203 ± 0.023 | 101 | 11.2 | 0.188 ± 0.009 | 94.2 | 4.61 |
|  | 2.7 | 2.89 ± 0.169 | 107 | 5.85 | 2.72 ± 0.227 | 101 | 8.35 |
|  | 58.3 | 60.5 ± 5.40 | 104 | 8.92 | 58.1 ± 6.40 | 99.6 | 11.0 |
| TSG | 0.3 | 0.304 ± 0.026 | 101 | 8.45 | 0.293 ± 0.019 | 97.5 | 6.65 |
|  | 0.6 | 0.639 ± 0.042 | 107 | 6.58 | 0.612 ± 0.042 | 102 | 6.87 |
|  | 8.1 | 7.73 ± 0.549 | 95.5 | 7.10 | 8.32 ± 0.330 | 103 | 3.97 |
|  | 175 | 181 ± 18.0 | 103 | 9.98 | 166 ± 11.1 | 94.9 | 6.69 |
| TG | 0.1 | 0.101 ± 0.009 | 101 | 9.03 | 0.102 ± 0.009 | 102 | 8.77 |
|  | 0.2 | 0.194 ± 0.008 | 96.7 | 3.95 | 0.211 ± 0.010 | 105 | 4.88 |
|  | 2.7 | 2.66 ± 0.199 | 98.5 | 7.50 | 2.82 ± 0.120 | 104 | 4.25 |
|  | 58.3 | 55.6 ± 1.87 | 95.3 | 3.37 | 56.2 ± 1.68 | 96.5 | 2.99 |
| CG | 0.1 | 0.101 ± 0.006 | 101 | 6.06 | 0.094 ± 0.008 | 94.1 | 8.70 |
|  | 0.2 | 0.210 ± 0.008 | 105 | 3.72 | 0.195 ± 0.017 | 97.7 | 8.66 |
|  | 2.7 | 2.56 ± 0.073 | 94.7 | 2.85 | 2.76 ± 0.258 | 102 | 9.36 |
|  | 58.3 | 58.0 ± 4.39 | 99.4 | 7.57 | 60.8 ± 4.45 | 104 | 7.32 |
| EG | 0.1 | 0.094 ± 0.005 | 93.8 | 5.22 | 0.102 ± 0.009 | 102 | 8.59 |
|  | 0.2 | 0.188 ± 0.014 | 94.2 | 7.53 | 0.195 ± 0.014 | 98.1 | 7.44 |
|  | 2.7 | 2.61 ± 0.118 | 96.6 | 4.52 | 2.84 ± 0.227 | 105 | 7.99 |
|  | 58.3 | 60.4 ± 1.97 | 104 | 3.27 | 62.8 ± 3.07 | 108 | 4.89 |
| PG | 0.1 | 0.102 ± 0.008 | 102 | 7.38 | 0.096 ± 0.009 | 96.3 | 9.69 |
|  | 0.2 | 0.210 ± 0.009 | 105 | 4.27 | 0.209 ± 0.012 | 105 | 5.79 |
|  | 2.7 | 2.79 ± 0.267 | 103 | 9.88 | 2.57 ± 0.134 | 95.3 | 4.95 |
|  | 58.3 | 54.3 ± 1.75 | 93.1 | 3.00 | 57.6 ± 3.39 | 98.9 | 5.81 |

**Supplemental dose-exposure relationship**

The dose-exposure relationship was evaluated by the power function model combined with the confidence interval method. First, the administration dose, AUC_0-∞_ and *C*_max_ were logarithmically transformed, and then linear regression was performed to obtain the curve correlation coefficient (R^2^), slope and 90% confidence interval (90% CI).

The active ingredient of RPM extract were directly proportional to the dose except for rhein and chrysophanol. The AUC_0-∞_ of chrysophanol, emodin, questinol, TSG and PG were nonlinearly related to the dose (90% CI of the slope were beyond the critical interval of 0.88–1.12), whereas the AUC_0-∞_ and dose of aloe-emodin, citreorosein, TG, EG showed uncertainty of linear relationship (90% CI of the slope of some samples were within the critical interval of 0.88–1.12). Additionally, it was observed that the *C*_max_ of emodin, TSG, TG, and PG were nonlinearly related to the dose (90% CI of the slope were beyond the critical interval of 0.80–1.20), whereas those of aloe-emodin, citreorosein, questinol, and EG could not be linearly correlated with the dose (90% CI of the slope of some samples were within the critical interval of 0.80–1.20). Understanding the pharmacokinetics of a drug that affect the drug availability at the target site is essential to elicit the effects of a drug on its target.

**Figure S5 (1)** Relationship between system exposure level (AUC_0-∞_and *C*_max_) of 10 constituents and p.o. dose of RPM extract in rats.

**Figure S5 (2)** Relationship between system exposure level (AUC_0-∞_and *C*_max_) of 10 constituents and p.o. dose of RPM extract in rats.

**Supplemental tissue distribution**

For the tissue distribution study, 18 rats were randomly divided into three groups (n=6, each), and orally administered with 18 g/kg RPM extract. The rats were sacrificed at 5, 15, 60 min after orally administering RPM. Subsequently, the heart, liver, spleen, lung, kidney, brain, stomach, small intestine, bladder, gonad (testes of male rats, uterus and ovaries of female rats) were immediately collected. An accurately weighed amount of fresh tissue sample (0.25 g) was individually homogenized with normal saline (1 mL) and transferred (50 μL) as a tissue homogenate to 1.5 mL centrifuge tubes for use as the tissue samples.

Compared with other organs, emodin and chrysophanol had the highest exposure in the spleen (AUC_0-t_ is 19100 and 50.3 nmol∙h/L), whereas the remaining seven compounds had the highest exposure in the liver tissue. Chrysophanol, aloe-emodin, and questinol ether were rarely exposed in the liver, which could be related to their lower content in the extract. The AUC_0-t_ of TSG in the kidney was 86400 nmol∙h/L, and the AUC_0-t_ of other compounds varied from 9.77 to 3210 nmol∙h/L. In addition, chrysophanol, aloe-emodin, and questinol ether were rarely exposed in the kidneys, which was also related to their lower content in the extract.

**Table S6** AUC_0-t_ (nmol·h/L) of 9 constituents in rat tissues.

| **Tissue** | **heart** | **liver** | **spleen** | **lung** | | **kidney** | **brain** | **stomach** | **intestine** | **bladder** | **testis** | **uterus** | **ovary** |
| --- | --- | --- | --- | --- | --- | --- | --- | --- | --- | --- | --- | --- | --- |
| Chrysophanol | 31.1 | 27.3 | 50.3 | 47.6 | 13.2 | | – | 34.5 | 27.7 | 37.6 | – | 29.0 | – |
| Emodin | 1640 | 6360 | 19100 | 7110 | 2830 | | 244 | 1720 | 5570 | 988 | 116 | 773 | 570 |
| Aloe-emodin | 16.9 | 55.5 | 52.5 | 31.3 | 9.77 | | 1.06 | 23.8 | 7.33 | – | – | – | 6.66 |
| Citreorosein | 650 | 1600 | 873 | 580 | 587 | | 5.83 | 783 | 211 | 60.4 | 13.7 | 104 | 227 |
| Questinol | 34.0 | 55.3 | 10.3 | 25.1 | 24.3 | | – | 6.10 | – | 16.9 | – | 18.6 | 16.8 |
| TSG | 45500 | 114000 | 68700 | 35100 | 86400 | | 6420 | 47900 | 71700 | 18200 | 7770 | 12200 | 7590 |
| TG | 1780 | 3810 | 1190 | 438 | 845 | | 18.5 | 1460 | 181 | 438 | 69.8 | 380 | 153 |
| EG | 914 | 2200 | 995 | 590 | 907 | | 58.7 | 238 | 483 | 212 | 298 | 439 | 634 |
| PG | 1640 | 3380 | 2690 | 842 | 3210 | | 125 | 1290 | 692 | 205 | 80.6 | 735 | 614 |

–: Not calculated.

**Supplemental Excretion study**

For the excretion study, 6 rats were placed in metabolic cage after orally administered with 18 g/kg RPM extract, and urine, feces samples were collected at time intervals of 0–4, 4–8, 8–24, 24–32, 32–48, 48–60, and 60–72 h. Another 6 rats were anesthetized with pentobarbital, fixed in the supine position and performed bile duct drainage operation, and the abdominal wound was sutured. The blank bile the state of the rats were weighed. Subsequently, they were orally administered with 18 g/kg RPM extract. The bile samples were collected at time intervals of 0–1, 1–2, 2–4, 4–6, 6–8, 8–24, 24–32 and 32–48 h. The urine, feces, and bile samples were stored at −70 °C until use.

The results show that most of RPM’s active ingredients can be excreted through urine excretion, bile secretion and secretion by intestinal epithelial cells. Among them, PG, rhein, and emodin show the possibilities to enter hepatic intestinal circulation, wherein TSG, chrysophanol, TG, and EG are mainly excreted in the form of intestinal epithelial cells and urine. The mass balance of each compound of RPM is shown in Table S8.

**Table S7** The cumulative excretion of dose excreted into urine, feces, and into bile of the 10 constituents in rat after single p.o. administration of RPM extract at 18 g/kg.

| **Compound** | **Urine(nmol)** | **Feces(nmol)** | **Bile(nmol)** |
| --- | --- | --- | --- |
| Chrysophanol | 13.2 ± 2.67 | 23.4 ± 4.76 | – |
| Emodin | 19.0 ± 3.81 | 50.3 ± 6.18 | 70.7 ± 20.4 |
| Aloe-emodin | 19.3 ± 5.44 | 43.7 ± 6.18 | 61.1 ± 8.66 |
| Rhein | – | – | 45.0 ± 8.41 |
| Citreorosein | 135 ± 23.4 | 202 ± 37.7 | 158 ± 13.2 |
| Questinol | – | 0.566 ± 0.0666 | – |
| TSG | 1619 ± 208 | 2778 ± 349 | 410 ± 55.4 |
| TG | 19.1 ± 2.99 | 30.4 ± 5.73 | 8.96 ± 2.38 |
| EG | 92.7 ± 31.0 | 342 ± 61.8 | 156 ± 21.9 |
| PG | 5.53 ± 2.08 | 13.6 ± 1.12 | 18.2 ± 3.29 |

–: Not calculated.

Table S8 The mass balance of each compound of RPM

| compound | Recovery in urine (%) | Recovery in feces (%) | Recovery in urine and feces (%) | Recovery in bile (%) |
| --- | --- | --- | --- | --- |
| Chrysophanol | 11.57 | 17.10 | 28.67 | – |
| Emodin | 8.82 | 23.64 | 32.46 | 33.64 |
| Aloe-emodin | 9.69 | 21.69 | 31.38 | 31.37 |
| Rhein | – | – | – | 37.79 |
| Citreorosein | 17.66 | 26.36 | 44.02 | 21.01 |
| Questinol | – | – | 4.12 | – |
| TSG | 14.30 | 24.56 | 38.86 | 3.71 |
| TG | 17.57 | 27.93 | 45.50 | 8.42 |
| EG | 7.76 | 28.56 | 36.32 | 13.34 |
| PG | 4.83 | 11.57 | 16.40 | 16.27 |

–: Not calculated.
